# A bioreducible *N*-oxide-based probe for photoacoustic imaging of hypoxia

**DOI:** 10.1038/s41467-017-01951-0

**Published:** 2017-11-27

**Authors:** Hailey J. Knox, Jamila Hedhli, Tae Wook Kim, Kian Khalili, Lawrence W. Dobrucki, Jefferson Chan

**Affiliations:** 1 Department of Chemistry, University of Illinois at Urbana-Champaign, 600 S. Mathews Ave, Urbana, IL 61801 USA; 2 Beckman Institute for Advanced Science and Technology, University of Illinois at Urbana-Champaign, 405 N. Mathews Ave, Urbana, IL 61801 USA; 3 Department of Bioengineering, University of Illinois at Urbana-Champaign, 1304 W. Springfield Ave, Urbana, IL 61801 USA

## Abstract

Hypoxia occurs when limited oxygen supply impairs physiological functions and is a pathological hallmark of many diseases including cancer and ischemia. Thus, detection of hypoxia can guide treatment planning and serve as a predictor of patient prognosis. Unfortunately, current methods suffer from invasiveness, poor resolution and low specificity. To address these limitations, we present Hypoxia Probe 1 (HyP-1), a hypoxia-responsive agent for photoacoustic imaging. This emerging modality converts safe, non-ionizing light to ultrasound waves, enabling acquisition of high-resolution 3D images in deep tissue. HyP-1 features an *N*-oxide trigger that is reduced in the absence of oxygen by heme proteins such as CYP450 enzymes. Reduction of HyP-1 produces a spectrally distinct product, facilitating identification via photoacoustic imaging. HyP-1 exhibits selectivity for hypoxic activation in vitro, in living cells, and in multiple disease models in vivo. HyP-1 is also compatible with NIR fluorescence imaging, establishing its versatility as a multimodal imaging agent.

## Abstract (web summary)

Hypoxia is a hallmark of many diseases including cancer and ischemia, and detection can be invasive and of low resolution and specificity. Here the authors show a hypoxia probe that converts non-ionizing light to ultrasound, which enables the acquisition of high-resolution 3D images in deep tissue.

## Introduction

Hypoxia occurs when tissue oxygen supply is restricted, leading to significant changes in oxygen-dependent physiological processes. The importance of proper oxygen regulation is evident from the variety of disease states associated with hypoxia, such as coronary and peripheral artery diseases^1^, alcoholic liver injury^2^, and gastrointestinal inflammatory conditions^3, 4^. Additionally, an estimated 50–60% of solid tumors contain hypoxic regions, which is noteworthy considering that hypoxic tumors are commonly associated with treatment resistance, aggressive phenotypes, and high metastatic potential^5^. Approximately 90% of breast cancer deaths occur as a result of metastasis, which is strongly promoted by increased intratumoral levels of hypoxia-inducible factors^6^. Rapid and specific hypoxia detection in vivo is therefore of great significance in both preclinical and clinical settings. Specifically, the ability to reliably and noninvasively detect hypoxia in real time can provide critical information to predict treatment responses and enable patient-specific treatment plans^7–10^.

The current gold standard of hypoxia detection is the oxygen-sensitive electrode, which is inserted into the tissue of interest to provide a direct measure of oxygen partial pressure. Although this technique enables oxygen detection with high sensitivity and accuracy, it is extremely invasive and therefore only applicable when tissue is accessible, such as in superficial tumors of the head and neck^10^. As such, significant effort has been put forth to develop alternative methods for noninvasive hypoxia detection. Optical imaging with hypoxia-responsive fluorescent probes has enabled visualization of hypoxia in living cells with excellent subcellular resolution^11–14^. However, due to high scattering and limited penetration of light in biological tissue, fluorescent probes suffer greatly from poor resolution beyond an imaging depth of 1 mm^15, 16^. Likewise, positron emission tomography (PET)-based hypoxia detection using various ^18^F-labeled radiotracers also faces major obstacles including high background due to non-specific uptake and limited spatial resolution^17, 18^. Together, these drawbacks hamper the ability to confidently discern specific hypoxic regions using PET imaging.

Photoacoustic (PA) imaging is a rapidly emerging modality that utilizes near-infrared (NIR) light from a pulsed laser source to induce temperature and pressure fluctuations in tissue, producing ultrasound waves that can be detected using acoustic transducers^19–21^. Because the scattering of sound in biological tissues is three orders of magnitude less than the scattering of light, these signals can be reconstructed to produce high-resolution 3D images (e.g., tens of microns) in deep tissue (e.g., cm range). In addition to superior resolution at these depths, PA imaging is a safer alternative compared to other techniques owing to the use of non-ionizing light. This property enables longitudinal tracking of disease progression without the risk of overexposure to harmful radiation^22^. Despite these advantages, there are currently no NIR-absorbing small-molecule PA imaging agents that allow for detection of specific tissue microenvironments via signal enhancement^23, 24^, although several peptide^25, 26^ and nanoparticle-based^27–31^ designs have been reported.

In this work, we present the development of Hypoxia Probe 1 (HyP-1), a hypoxia-responsive agent for PA imaging. HyP-1 features an *N*-oxide-based trigger that can undergo facile bioreduction in the absence of oxygen. This design was inspired by AQ4N, a hypoxia-responsive cancer prodrug containing two *N*-oxide moieties that mask its cytotoxic effects prior to reduction in hypoxic tissue^32–34^. Although *N-*oxides have been employed for fluorescence sensing of Fe(II)^35, 36^, bioorthogonal conjugation reactions^37^ and flow cytometric analysis of apoptotic cell populations^38^; this functionality has not previously been demonstrated for specific hypoxia detection. The PA response of HyP-1 relies on the ability of the *N*-oxide to modulate its optical properties. Specifically, conversion of the *N*-oxide to the corresponding aniline (red-HyP-1) elicits a bathochromic (red) shift in the absorbance from 670 to 760 nm. Because HyP-1 does not absorb at this wavelength, the PA signal produced upon excitation at 760 nm corresponds exclusively to red-HyP-1. Therefore, hypoxia detection is made possible by determining the signal enhancement observed at this wavelength. Additionally, because both HyP-1 and red-HyP-1 are fluorescent, HyP-1 can be employed for ratiometric NIR fluorescence imaging in both living cells and animal models. Rapid and selective hypoxia-mediated activation of HyP-1 is observed both in vitro and in cancer cells cultured under hypoxic conditions. HyP-1 can be applied to hypoxia detection in vivo, which we demonstrate with the application of a hypoxic tumor model. Importantly, HyP-1 is unlike many cancer-specific hypoxia imaging probes that rely on enzyme upregulation for activation to occur. In addition to intratumoral hypoxia, HyP-1 can also reliably detect oxygen deficiencies in vivo in a hindlimb ischemia model of peripheral artery disease (PAD). The success of HyP-1 in these different disease models is indicative of its versatile capabilities and applicability to hypoxia detection.

## Results

### Design and synthesis

**Fig. 1:**
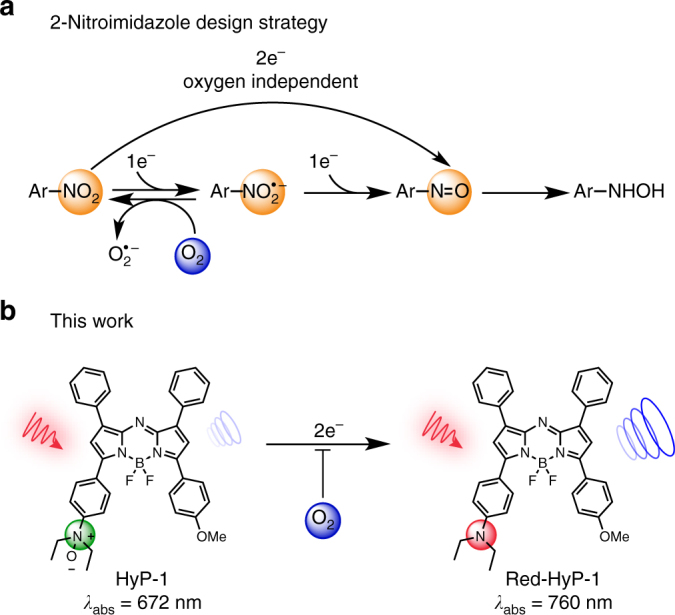
Design strategies used for hypoxia-responsive probe development. **a** Enzymatic reduction of 2-nitroimadazoles. One-electron reductases reduce the nitro to the corresponding nitro radical anion, which can be rapidly reversed by oxygen. In the absence of oxygen, a subsequent reduction affords the nitroso, which can then undergo further reduction and irreversible crosslinking with intracellular nucleophiles. **b** Chemical structure of HyP-1 and red-HyP-1. HyP-1 undergoes irreversible two-electron reduction by heme proteins such as CYP450 enzymes in the absence of oxygen, which binds competitively to the heme iron. Red-HyP-1 produces an enhanced PA signal (blue circles) upon irradiation at 770 nm (red arrow)

The development of hypoxia-responsive imaging probes has focused primarily on the use of the 2-nitroimidazole moiety^39^. This functionality has been applied to numerous fluorescence-based probes as well as several ^18^F- and ^99^Tc-labeled radiotracers for PET and SPECT, respectively^10, 40–42^. The mechanism of activation for these compounds relies on two subsequent one-electron reductions performed by nitroreductases. The first of these reductions produces a nitro radical anion, which can be rapidly reoxidized to the parent nitro compound by molecular oxygen. However, in the absence of oxygen, the nitro radical anion can undergo further reduction to form reactive intermediates that are susceptible to attack by intracellular nucleophiles, resulting in irreversible crosslinking (Fig. 1a). Despite the many examples of this strategy for hypoxia targeting, its application for PA probe design faces several drawbacks. First, the various reduced intermediates that form can have distinct PA properties, which may produce ambiguities in the signal detected. Second, although one-electron reductases are primarily responsible for the reductive activation of these compounds, reduction can also occur via two-electron pathways in an oxygen-independent manner, resulting in false-positive signals^43^. Oxygen-independent reduction can also be achieved by bacterial nitroreductases and analogous enzymes found in the mitochondria, providing additional sources of non-specific activation^44^. Finally, reductive activation of 2-nitroimidazole compounds is most commonly associated with chronic rather than acute hypoxia, limiting its application to certain models^39^.

The *N*-oxide-based trigger of HyP-1 presents a unique, alternative strategy. First reported by Sugiura et al. in 1977, the mechanism of oxygen dependency for the bioreduction of *N*-oxides relies on competitive binding of oxygen to the heme iron of various CYP450 enzymes^32, 45, 46^. In the absence of oxygen, the *N*-oxide can bind and undergo irreversible two-electron reduction. This design therefore precludes redox cycling and formation of various intermediates, thus minimizing false positives. In addition to a hypoxia-responsive trigger, our PA probe design requires several other important considerations such as high absorptivity, absorbance in the NIR window (650–900 nm), and extensive photostability. The aza-BODIPY dye platform has been shown in our recent work to possess these key features^23^. For the design of HyP-1, we chose to use an unsymmetric aza-BODIPY containing our hypoxia-responsive trigger on one side and a methoxy substituent on the other (Fig. 1b).

**Fig. 2:**
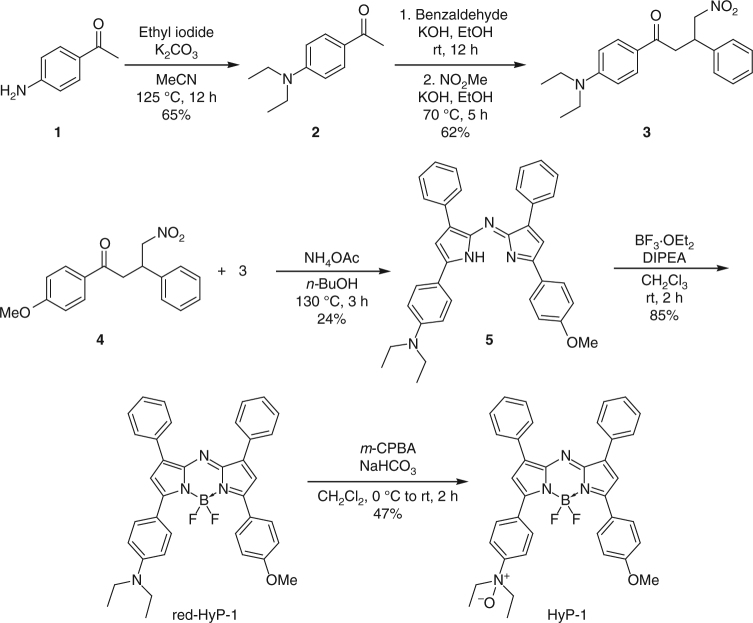
Synthesis of HyP-1 and red-HyP-1. Red-HyP-1 was obtained via heterodimerization of key precursors **3** and **4**, followed by BF_2_ incorporation using BF_3_·OEt_2_. HyP-1 was obtained via oxidation of red-HyP-1 using *m*-CPBA

As shown in Fig. 2, the synthesis of HyP-1 began with the preparation of key building blocks **3** and **4**. This was accomplished via Claisen–Schmidt condensation of benzaldehyde and the corresponding acetophenone precursor, followed by 1,4-addition of the nitromethane anion. Heterodimerization of **3** and **4** provided tetraarylazadipyrromethene **5** in 24% yield. Compound **5** was then treated with BF_3_·OEt_2_ in the presence of diisopropylethylamine to form the aza-BODIPY core of red-HyP-1 in 85% yield. Finally, oxidation of the aniline to the *N*-oxide with *m*-CPBA afforded HyP-1 in 47% yield.

### Photophysical characterization

**Fig. 3:**
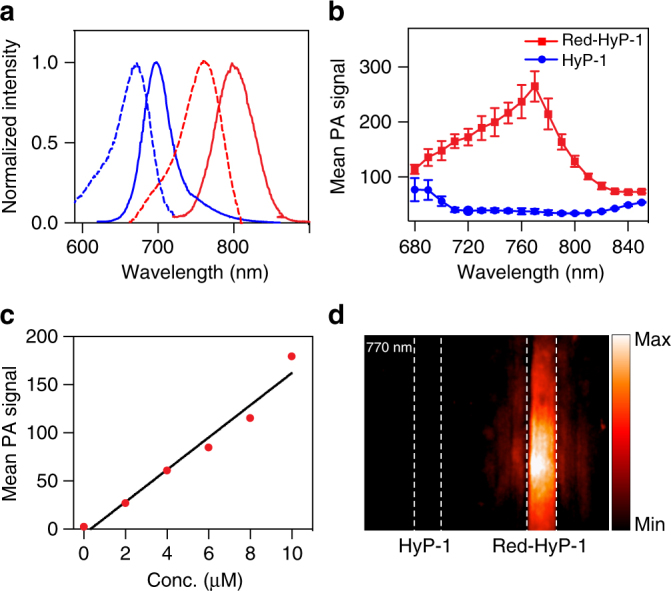
Photophysical characterization of HyP-1 and red-HyP-1. **a** Normalized absorbance (dashed) and emission (solid) spectra of HyP-1 (blue) and red-HyP-1 (red). **b** PA spectra of HyP-1 and red-HyP-1. PA images of HyP-1 and red-HyP-1 solutions (10 μM in 0.1 M potassium phosphate buffer (pH 7.4) with 50% EtOH co-solvent) in tissue-mimicking phantoms were obtained, and the mean PA signal corresponding to each compound was plotted as a function of wavelength. Results are presented as mean ± SD (*n* = 3). **c** PA signal corresponding to various concentrations of red-HyP-1. **d** PA image (770 nm) of HyP-1 and red-HyP-1 solutions (10 μM in 0.1 M potassium phosphate buffer with 50% EtOH co-solvent) in tissue-mimicking phantom. Dashed lines indicate positioning of FEP tubes

Having established an effective synthetic route, we turned our attention to the photophysical characterization of HyP-1 and red-HyP-1 (Supplementary Table 1). HyP-1 has an absorbance maximum at 670 nm and corresponding emission maximum at 697 nm (Fig. 3a). The extinction coefficient (*ε*) and quantum yield (Φ_F_) were measured to be 1.5 × 10^4^ cm^−1^ M^−1^ and 0.33, respectively. Red-HyP-1, on the other hand, has absorbance and emission maxima at 760 nm and 798 nm, respectively. Importantly, red-HyP-1 exhibits an increase in the extinction coefficient (*ε* = 5.4 × 10^4^ cm^−1^ M^−1^) and decrease in quantum yield (Φ_F_ = 0.15), both of which favor an enhanced PA signal (vide infra).

The significant change in the absorbance profiles of these compounds can be attributed to disruption of the electron-donating capability of the aniline in its *N*-oxide form. This chemical modification is akin to protonation of the aniline, which has been previously shown to result in similar shifts in the absorbance^47^. The 90 nm separation in absorbance is essential to our probe design as it allows us to readily distinguish each species by selective excitation and subsequent detection using both PA and fluorescence imaging. Indeed, the PA spectra recorded for both HyP-1 and red-HyP-1 correlate well with their absorbance profiles and demonstrate that a strong PA signal can be detected from red-HyP-1 at 770 nm in a concentration-dependent manner, while no signal is produced by HyP-1 at this wavelength (Fig. 3). Additionally, because HyP-1 and red-HyP-1 have spectrally resolved emission profiles, ratiometric imaging can be performed by determining the ratio of their fluorescence emission intensities. Ratiometric imaging can account for common imaging artefacts such as uneven dye loading and photobleaching, making this a highly attractive feature for both cellular and in vivo imaging.

### In vitro characterization of HyP-1

**Fig. 4:**
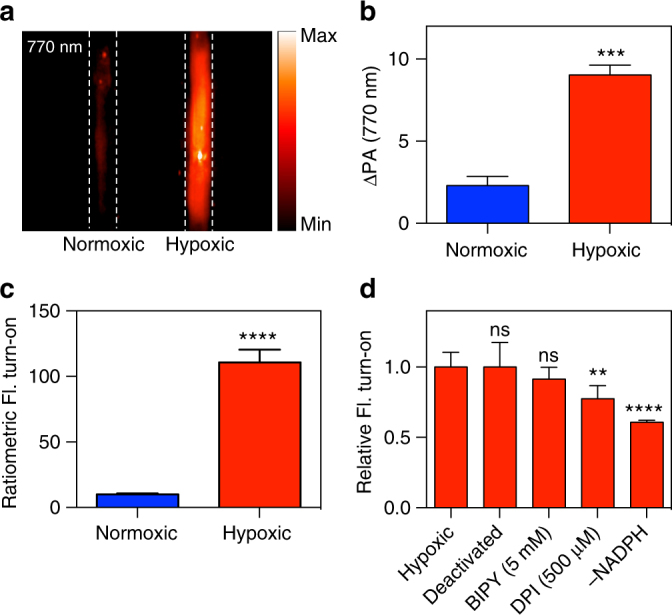
Hypoxia-selective response of HyP-1 in vitro. **a** Representative PA image (770 nm) of reaction mixtures incubated under normoxic or hypoxic conditions for 2 h, then quenched with an equal volume of acetonitrile. Dashed lines indicate positioning of FEP tubes. **b** Change in PA signal (770 nm) produced after 2 h incubation of HyP-1 (10 μM) with rat liver microsomes (RLM) (200 μg/mL) and NADPH (50 μM) in normoxic and hypoxic conditions (*n* = 3). **c** Ratiometric fluorescence turn-on after 2 h incubation of HyP-1 (2 μM) with RLM (200 μg/mL) and NADPH (50 μM) in normoxic or hypoxic conditions (*n* = 3). **d** Normalized fluorescent turn-on after 1 h hypoxic incubation of HyP-1 (2 μM) with RLM (100 μg/mL) and NADPH (50 μM) under various conditions. Statistical significance represents a comparison to the hypoxic control (*n* = 6). Results with error bars are represented as mean ± SD. **p < 0.01, ***p < 0.001, ****p < 0.0001

The responsiveness of HyP-1 to hypoxia was first evaluated in vitro via fluorescence by incubating HyP-1 with CYP450-rich rat liver microsomes under atmospheric (normoxic) and oxygen-deprived (hypoxic) conditions. Rat liver microsomes are most commonly employed to assess drug metabolism, but have also been extensively used to study the activation of hypoxia-responsive probes and prodrugs^11–13, 34^. Under hypoxic conditions, a time-dependent fluorescence enhancement at 785 nm and concomitant decrease at 710 nm was observed, indicating complete conversion of HyP-1 to red-HyP-1 with no apparent formation of by-products (Supplementary Fig. 1). In particular, an authentic solution of red-HyP-1 is identical in appearance (red in color) and exhibits an emission maximum (785 nm) indistinguishable from that of the RLM reaction mixture. This was further confirmed by mass spectrometry analysis, in which an M + 1 peak of 599.2817 corresponding to red-HyP-1 was observed (Supplementary Fig. 2). Overall, an average 105-fold ratiometric turn-on response occurred after hypoxic incubation for 2 h at 37 °C, while only a small 10-fold change in the ratiometric fluorescence resulted from normoxic incubation (Fig. 4c). The PA response of HyP-1 under identical conditions was evaluated by quenching the reaction mixtures with an equal volume of acetonitrile and transferring the solutions into fluorinated ethylene propylene (FEP) tubes, which were inserted into the center of a 2 cm-thick agarose-based tissue-mimicking phantom. Irradiation at 770 nm afforded a fourfold greater PA signal produced from the hypoxic reaction (Fig. 4a, b).

Fe(II)-mediated reduction of *N*-oxides has been exploited previously for the development of several fluorescent probes for this metal ion^35, 36^, thus it was critical for us to determine the response of HyP-1 to Fe(II). HyP-1 exhibited a mere eightfold ratiometric turn-on after a 1-h incubation in the presence of 20 μM FeSO_4_ in HEPES buffer (Supplementary Fig. 3). A more significant turn-on response of 35-fold (one-third of the maximum turn-on) was observed when the solution was supplemented with 1 mM GSH, indicating that Fe(II) is capable of converting HyP-1 to red-HyP-1 under these conditions. However, because intracellular concentrations of free iron are low due to sequestration by ferritin^48^, we did not anticipate this to be a major source of HyP-1 activation in vivo.

We hypothesized that the reduction of HyP-1 by hypoxic rat liver microsomes is due to heme-based redox proteins, such as CYP450 enzymes, a process that has been previously reported for various *N*-oxide-containing compounds^32, 46, 49, 50^. However, it has also been shown that Fe(II) can be released from ferritin in rat liver microsomes under anaerobic conditions^51, 52^, and given the response of HyP-1 to Fe(II), we sought to verify the factors responsible for HyP-1 reduction in vitro. When an excess of 2,2′-bipyridine (BIPY), an iron chelator, was added to HyP-1 in the presence of rat liver microsomes under hypoxic conditions, no significant inhibition of the turn-on response was observed (Fig. 4d). In contrast, inhibition of HyP-1 reduction was achieved both by the addition of diphenyliodonium chloride (DPI), an inhibitor of CYP450 reductases^53^, and exclusion of NADPH, which is necessary for enzymatic activity. Interestingly, HyP-1 was also converted to red-HyP-1 in the presence of heat-inactivated rat liver microsomes in a manner similar to several previously reported *N*-oxide-containing compounds^49, 50^. These data led us to conclude that microsomal reduction of HyP-1 is independent of labile Fe(II) and relies instead upon both enzymatic and non-enzymatic bioreduction by the heme iron of CYP450 enzymes, as well as other heme-based redox proteins.

In addition to Fe(II), we also determined the response of HyP-1 to a variety of alkali, alkaline earth, and transition metals. No significant turn-on was observed, verifying the stability of HyP-1 under these experimental conditions (Supplementary Fig. 4). Likewise, treatment of HyP-1 with a variety of reactive oxygen, nitrogen, and sulfur species commonly encountered in the cellular environment yielded similar results (Supplementary Fig. 5). To further evaluate the stability of HyP-1, a 10 μM solution of the probe was exposed to ambient light and temperature for up to 5 days. In addition, a 10 μM solution of HyP-1 supplemented with human plasma was incubated at 37 °C for 3 h. During these experiments, no substantial decomposition of HyP-1 was observed, demonstrating its exceptional chemostability (Supplementary Figs. 6 and 7). Furthermore, the absorbance and emission spectra of HyP-1 and red-HyP-1 in buffers ranging in pH from 4.8 to 12.0 revealed no significant pH dependence (Supplementary Figs. 8 and 9). As mentioned previously, a major limitation of 2-nitroimidazole-based hypoxia probes is their susceptibility to oxygen-independent reduction via two-electron processes, such as by bacterial nitroreductases. When HyP-1 was incubated with either purified bacterial nitroreductase or *E. coli* cell lysates, minimal conversion to red-HyP-1 was observed (Supplementary Fig. 10). Finally, the photostability of both HyP-1 and red-HyP-1 was evaluated via continuous PA image acquisition at 770 nm over the course of 1 h. The PA intensities of both sample solutions remained unchanged during this time, demonstrating the excellent photostability of both compounds (Supplementary Fig. 11).

### Detecting hypoxia in living cells with HyP-1

**Fig. 5:**
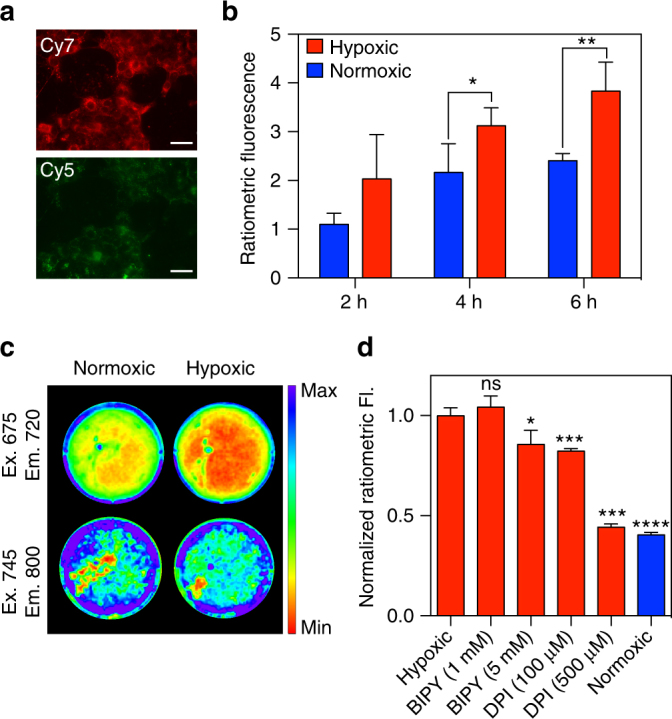
Ratiometric fluorescence imaging of HyP-1 in hypoxic cell culture. **a** Representative images (scale bar represents 50 μm) and **b** quantification of time-dependent ratiometric fluorescence of 4T1 cells stained with 5 μM HyP-1 in serum-free medium and incubated under hypoxic or normoxic conditions. Ratios determined by dividing Cy7 emission by Cy5 emission (*n* = 4). **c** IVIS spectrum images of 4T1 cells stained with HyP-1 (5 μM) and incubated under normoxic or hypoxic conditions for 4 h. **d** Normalized ratiometric fluorescence of 4T1 cells stained with HyP-1 (5 μM) under various hypoxic (red) or normoxic (blue) conditions. Statistical significance represents a comparison to the hypoxic control. Results with error bars are represented as mean ± SD. *p < 0.05, **p < 0.01, ***p < 0.001, ****p < 0.0001

After establishing excellent selectivity and responsiveness to hypoxic conditions in vitro, we sought to evaluate the performance of HyP-1 in living cells before moving to in vivo studies. 4T1 murine mammary carcinoma cells were treated with a 5 μM solution of HyP-1 and incubated at 37 °C either in a standard atmosphere containing 20% oxygen (normoxic conditions), or in an airtight chamber containing < 0.1% oxygen (hypoxic conditions). Imaging was performed at 2, 4, and 6 h using an epi-fluorescence microscope equipped with Cy5 and Cy7 filter sets to visualize HyP-1 and red-HyP-1, respectively (Fig. 5a). Cells incubated under hypoxic conditions exhibited a time-dependent ratiometric fluorescence response that was 1.6-fold higher than the normoxic control after 6 h (Fig. 5b).

Ratiometric fluorescence imaging was also performed on 4T1 cells cultured in six-well plates using an IVIS spectrum imaging system. Cells were stained with a 5 μM solution of HyP-1 and incubated in either normoxic or hypoxic conditions for 4 h (Fig. 5c). The ratiometric response of the hypoxic wells was found to be 2.5-fold higher than the normoxic response (Fig. 5d). To further verify the in vitro results and determine the cellular factors responsible for HyP-1 reduction, cells were also stained with HyP-1 solutions containing either BIPY or DPI at multiple concentrations and incubated under hypoxic conditions. The presence of 1 mM BIPY had no effect on the cellular response to HyP-1, while 5 mM BIPY resulted in only slight reduction of the ratiometric fluorescence. On the other hand, incubation with DPI resulted in significant dose-dependent inhibition, again implicating the role of heme-based redox proteins such as CYP450 enzymes in the reduction of HyP-1 (Fig. 5d and Supplementary Fig. 12).

### In vivo imaging of a hypoxic tumor-bearing mouse model

**Fig. 6:**
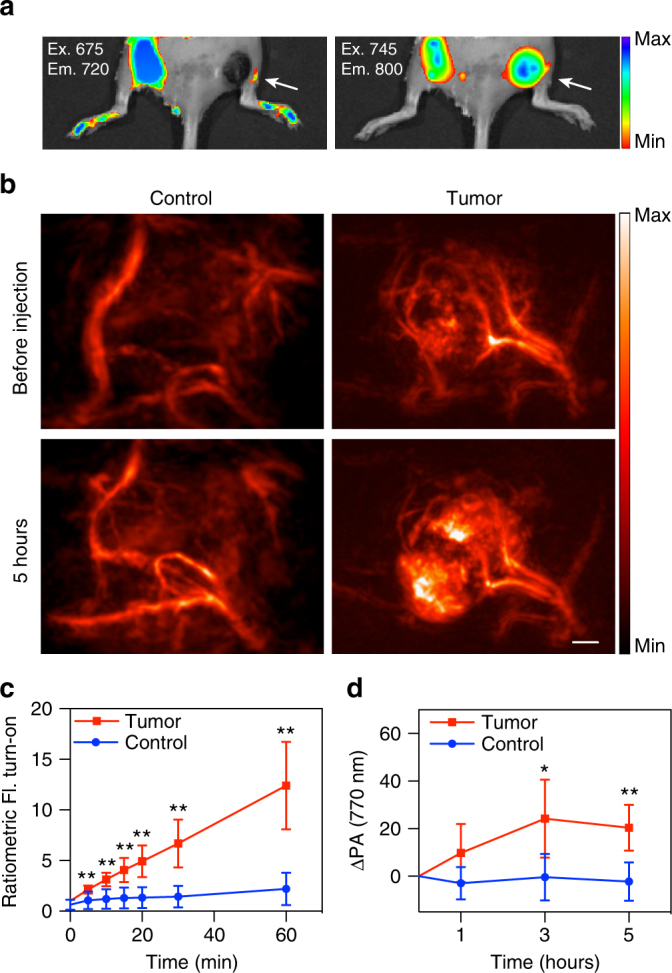
In vivo imaging of tumor hypoxia with HyP-1. **a** Fluorescence (IVIS spectrum) images acquired of a 4T1 tumor-bearing mouse 1 h following intratumoral injection (50 μL, 100 μM) of HyP-1. Images were acquired using 675/720 nm and 745/800 nm filter sets. Arrows indicate tumor. **b** PA images (770 nm) of the tumor-bearing and control flank before and 5 h following injection of HyP-1 (50 μL, 0.3 mg/kg). Scale bar represents 2 mm. **c** Time-dependent ratiometric fluorescence increase of tumor-bearing and control flanks (*n* = 5). **d** Time-dependent increase of PA signal of tumor-bearing and control flanks (*n* = 5). Results with error bars are represented by mean ± SD. *p < 0.05, **p < 0.01

To evaluate the utility of HyP-1 for visualization of intratumoral hypoxia, we employed a murine mammary carcinoma tumor model that has been previously utilized for evaluating hypoxia-responsive probes^54^. Tumor allografts were generated via subcutaneous implantation of 4T1 cells into the right flanks of BALB/c mice. The tumors were allowed to grow to volumes of 300–400 mm^3^ prior to imaging. As an initial study to visualize the intratumoral conversion of HyP-1 to red-HyP-1 in real time, ratiometric fluorescence imaging was performed immediately following administration of HyP-1 (50 μL, 100 μM) via intratumoral or subcutaneous (control) injection. After 1 h, the average ratiometric fluorescent turn-on was 5.6-fold greater in the tumor tissue, demonstrating the hypoxia-selective response of HyP-1 (Fig. 6a, c).

We next turned our attention to the application of HyP-1 for hypoxia detection using PA imaging. HyP-1 (50 μL, 0.3 mg/kg) was administered to tumor-bearing mice intravenously via tail vein injection, and PA images of both the tumor-bearing and control flanks were acquired at 1, 3, and 5 h following injection. An average mean PA signal increase of 20.5 (a.u.) was observed in the tumor tissue, while no increase was observed in the control flank (Fig. 6b, d). The 3D reconstructions of the PA images enabled visualization of specific areas within the tumor where signal was highest, a significant improvement compared to the minimally resolved signal obtained using fluorescence imaging (Fig. 6b and Supplementary Movie 1). We hypothesize that these areas of high intensity correlate with the highest concentrations of red-HyP-1, and therefore with regions of most severe hypoxia. These results demonstrate the superior resolution achievable in deep tissue using PA imaging, as well as the utility of HyP-1 for hypoxia detection following systemic administration.

### In vivo imaging of a murine hindlimb ischemia model

**Fig. 7:**
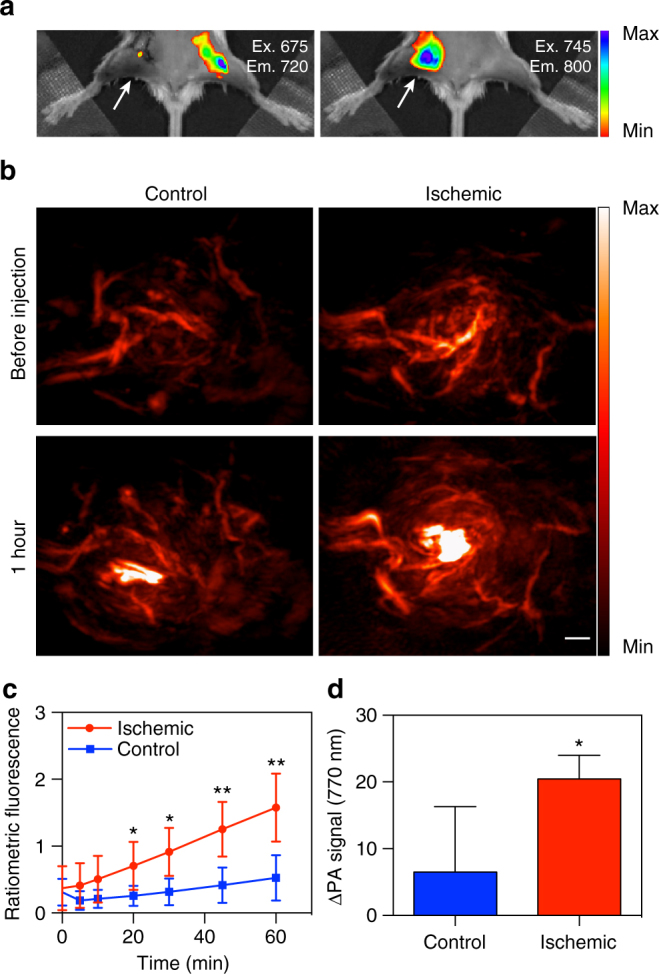
In vivo imaging of a murine hindlimb ischemia model with HyP-1. **a** Fluorescence (IVIS spectrum) images acquired of hindlimb ischemia mouse model (right leg) 1 h following intramuscular injection (50 μL, 50 μM) of HyP-1 in both legs. Images were acquired using 675/720 nm and 745/800 nm filter sets. Mouse imaged in supine position, arrows indicate ischemic tissue. **b** PA images (770 nm) of the ischemic and control leg before and 1 h following injection of HyP-1 (50 μL, 50 μM). Scale bar represents 2 mm. **c** Time-dependent ratiometric fluorescence increase of ischemic and control limbs (*n* = 5). **d** Time-dependent increase of PA signal of ischemic and control limbs (*n* = 4). Results with error bars are represented as mean ± SD. *p < 0.05, **p < 0.01

To visualize the response of HyP-1 in a model of rapidly developing hypoxia, we employed a murine hindlimb ischemia model of peripheral artery disease. Ischemia was induced in the right hindlimb of BALB/c mice via ligation of the femoral artery^55^. To confirm that the ligation had successfully limited blood perfusion through the limb, laser Doppler perfusion imaging was performed immediately following the surgery, and a drastic decrease in blood flow was observed in the right leg (Supplementary Fig. 13). After a 1 h recovery period, HyP-1 (50 μL, 50 μM) was administered via intramuscular injection into both hindlimbs of the mouse. To visualize the time-dependent response of HyP-1 in the ischemic and non-ischemic tissue, ratiometric fluorescence imaging was performed over the course of 1 h immediately following injection. A rapid increase in the ratiometric fluorescence intensity corresponding to conversion of HyP-1 to red-HyP-1 was observed in the ischemic tissue. Overall, the average ratiometric signal was threefold greater in the ischemic tissue compared to the control, indicating oxygen-dependent inhibition of HyP-1 activation (Fig. 7a, c). Moreover, we confirmed that the activation of HyP-1 under ischemic conditions does not involve Fe(II) by co-administering HyP-1 with BIPY (5 mM) to chelate free Fe(II). The ratiometric fluorescence enhancement was statistically indistinguishable from that of when HyP-1 was administered alone (Supplementary Fig. 14).

After observing the hypoxia-mediated response of HyP-1 in the hindlimb ischemia model, we turned our attention to detecting this response using PA imaging. As described above, surgical ligation was employed to induce ischemia, and HyP-1 (50 μL, 50 μM) was administered via intramuscular injection. PA images were acquired 1 h following injection, and a robust signal enhancement 3.1-fold greater on average than that of the control leg was observed in the ischemic tissue (Fig. 7b, d). These results demonstrate that HyP-1 can be applied to hypoxia detection in multiple disease models. In addition, the limited time frame between induction of ischemia and imaging suggests that HyP-1 does not rely significantly on upregulation of heme-based redox proteins, but rather can undergo rapid conversion to red-HyP-1 under hypoxic conditions at constitutive enzyme expression levels^56^.

## Discussion

As described above, we have designed and evaluated a hypoxia-responsive imaging agent for PA imaging. HyP-1 features a hypoxia-responsive trigger that enables rapid and direct conversion of an *N*-oxide to the corresponding aniline, preventing formation of intermediates that may introduce signal ambiguities^45^. Because this design relies primarily on competitive binding of oxygen rather than oxygen-dependent redox cycling, minimal background results from oxygen-independent reduction pathways. As such, this strategy presents a unique alternative for the design of various hypoxia-responsive probes, and we anticipate that this example will inspire many applications of this unique design strategy for hypoxia detection.

The design of small-molecule PA probes relies on the ability of the activated probe to produce a detectable PA signal enhancement compared to the unactivated probe. This can occur via one of several mechanisms, including increasing molar absorptivity, thus increasing the amount of incident light absorbed, decreasing quantum yield, resulting in a greater proportion of non-radiative decay or shifting the absorbance such that irradiation at a certain wavelength produces signal from only the activated probe. Hypoxia-mediated activation of HyP-1 facilitates a turn-on response using all three of these mechanisms. Specifically, conversion of HyP-1 to red-HyP-1 results in a 3.6-fold increase in extinction coefficient, a 2.2-fold decrease in quantum yield and a 90 nm shift in absorbance. Together, these features enable hypoxia detection using PA imaging with high sensitivity and reliability with minimal background.

Although HyP-1 was designed primarily for PA imaging, its NIR absorbance and emission profiles enable ratiometric NIR fluorescence imaging of hypoxia in cellular systems with fluorescence microscopy, followed by direct translation to in vivo models. The absorbance of endogenous chromophores (e.g., hemoglobin) is minimized within the NIR window, thus the development of NIR-absorbing probes enables maximum light penetration using optical imaging methods. Therefore, HyP-1 and red-HyP-1 can both be easily visualized in vivo using whole-body NIR fluorescence imaging. Although minimal resolution is achieved, this capability of HyP-1 renders it a highly convenient tool for rapid hypoxia detection in preclinical animal models.

Intratumoural hypoxia is a characteristic property of advanced solid tumors and is a key factor associated with increased metastatic potential and poor treatment outcomes^57, 58^. Tumor hypoxia results primarily from the inability of oxygen to diffuse to poorly vascularized areas (diffusion-limited) or temporary obstructions in blood flow that limit oxygen delivery (perfusion-limited). The heterogeneity of solid tumors can result in significant variations in oxygen deficiency from region to region, and although methods for noninvasive hypoxia detection exist, limited resolution and sensitivity produce significant challenges in determining the distribution of hypoxic volumes within a tumor^17^. We have demonstrated that PA imaging with HyP-1 enables 3D visualization of intratumoral hypoxia with excellent resolution. Comparison of PA images acquired before and after HyP-1 administration reveals specific regions of signal enhancement, which we propose to correlate to regions of the most severe hypoxia. The superior resolution and imaging depth of PA images compared to fluorescence images indicates the promising outlook of this emerging imaging modality.

PAD is a debilitating condition that affects nearly 10 million individuals in the United States^59^. PAD is characterized by restricted arterial circulation to the limbs, most commonly the legs, resulting from plaque deposition and arterial hardening. The murine hindlimb ischemia model is widely accepted for the study of PAD and has been used in the evaluation of potential treatment options. Due to the rapid oxygen depletion that results from this model, we envisioned it could be used to demonstrate the response of HyP-1 to hypoxic conditions in vivo independent of extensive changes in gene and protein expression levels that can result from prolonged hypoxia in other models (e.g., cancer)^60^. Indeed, we have demonstrated that HyP-1 exhibits a rapid turn-on response in murine hindlimb ischemia just 1 h following surgical ligation of the femoral artery. These results show the excellent sensitivity of HyP-1 to oxygen-deficient conditions resulting from ischemia. PAD represents just one of many ischemic conditions in which HyP-1 could be used to detect rapidly developing hypoxia.

Prior to this work, hypoxia detection using optical methods has relied extensively on fluorogenic probes that provide minimal resolution and depth penetration. Through the development of HyP-1, we have established criteria for employing optical-based probe designs for translation into powerful PA imaging agents. Probes, such as HyP-1, that undergo reaction-based activation to elicit a PA turn-on response can provide a target-specific readout in deep tissue, a direction that was previously exploited only for cell-based fluorescence imaging. While impressive, the advances in PA techniques and instrumentation have been constrained by a shortage of PA probes that can demonstrate target-specific detection. As such, the importance of HyP-1 extends beyond hypoxia detection; rather, we anticipate this work will stimulate further investigations in this rapidly growing arena.

## Methods

### Hypoxia sensing in vitro

For hypoxic assays only, cuvettes were sealed with septa, then evacuated and filled with N_2_ (×3) prior to use. Potassium phosphate buffer (0.1 M, pH 7.4) and NADPH (0.5 mM) were degassed with N_2_ for 30 min. For all assays, phosphate buffer (889 μL) rat liver microsomes (200 μg/mL) and HyP-1 (1 μL, 2 mM in DMSO) were added to the cuvette and pre-incubated at 37 °C for 5 min. NADPH (100 μL) was added and incubation continued with fluorescence spectra acquisition occurring at the indicated time points. Spectra were acquired according to the following parameters: *λ*_ex_ = 672, emission range = 685–800 nm and *λ*_ex_ = 750, emission range = 760–900 nm. For BIPY and DPI experiments, the compounds were added from stock solutions (1 M BIPY in DMSO and 50 mM DPI in deionized water) following the 5 min preincubation, and the buffer volume was adjusted such that the total volume in the cuvette was equal to 1 mL. Deactivated microsomes were prepared by heating in a 45 °C water bath for 30 min.

### Cell culture

Authenticated 4T1 cells were purchased from ATCC. Cells were grown in RPMI 1640 medium supplemented with 10% fetal bovine serum. Cells were cultured at 37 °C in 5% CO_2_ and 20% O_2_ for normoxic conditions. Hypoxic cell culture was performed by incubating cells in a sealed container with a Mitsubishi AnaeroPack™ anaerobic gas generator. Hypoxic conditions were verified with the use of a Mitsubishi RT Anaero-Indicator.

### Fluorescence imaging in living cells

For fluorescence microscopy, cells were plated in four-well chamber slides at an initial density of 1 × 10^4^ cells/mL and allowed to grow until 60–80% confluent (~6 days). Media was removed and cells were washed with PBS. A 5 μM solution of HyP-1 in serum-free medium was added, and cells were then incubated under hypoxic or normoxic conditions. Images were obtained using Cy5 and Cy7 filter cubes and analyzed using ImageJ software (Version 1.50i).

For cellular imaging using the IVIS spectrum imaging system, cells were plated in a six-well plate at an initial density of 1.7 × 10^4^ cells/mL and allowed to grow until 60–80% confluent (~3 days). Media was removed and cells were washed with PBS. A 5 μM solution of HyP-1 in serum-free medium was added, and cells were incubated under normoxic or hypoxic conditions for 4 h. For BIPY and DPI experiments, the staining solution was supplemented with the compound at the appropriate concentration, and ratiometric turn-on was reported relative to a vehicle control. Images were obtained using excitation and emission filter sets of 675/720 nm and 745/800 nm. Regions of interest (ROIs) were drawn around cell-containing wells, and ratiometric signal was calculated by determining the ratio of radiant efficiency corresponding to 800 and 720 nm emissions.

### Murine tumor model

All in vivo imaging experiments were performed with the approval of the Institutional Animal Care and Use Committee of the University of Illinois at Urbana-Champaign, following the principles outlined by the American Physiological Society on research animal use. Female mice (5–6 weeks old) were acquired from The Jackson Laboratory. A suspension of 0.5 × 10^6^ cells in serum-free medium containing 50% v/v Matrigel (100 μL) was subcutaneously injected into the right flank of each mouse. Tumors with volumes of 300–400 mm^3^ formed after 12–15 days. Animals with tumor volumes beyond this range were excluded from analysis.

### Murine ischemia model

All in vivo imaging experiments were performed with the approval of the Institutional Animal Care and Use Committee of the University of Illinois at Urbana-Champaign, following the principles outlined by the American Physiological Society on research animal use. Female mice (5–6 weeks old) were acquired from The Jackson Laboratory. Mice were anesthetized with isoflurane, and unilateral hindlimb ischemia was surgically induced by femoral artery ligation following previously published approaches^55, 61^. Animals that underwent unsuccessful surgery as determined by laser Doppler perfusion imaging were excluded from analysis.

### Fluorescence imaging in vivo

Images were obtained using epi-fluorescence with excitation filters of 675 and 745 and corresponding emission filters of 720 and 800, respectively. Data was processed using Living Image software (Version 4.1). ROIs of equal area were drawn around signals, and the total radiant efficiency in each ROI was determined. Ratiometric signal was calculated by determining the ratio of radiant efficiency corresponding to 800 and 720 nm emissions.

### PA imaging in tissue-mimicking phantoms

Tissue phantoms were prepared by suspending agarose (4 g) in a solution of 2% milk (2 mL) and deionized H_2_O (78 mL). The suspension was then heated in a microwave oven until a viscous gel was produced (30–50 s). The gel was transferred to a mold made by drilling holes through a 50 mL centrifuge tube and placing FEP tubes (0.08″ diameter) through the holes to create spaces for samples. After cooling the gel for a minimum of 3 h, the solidified phantom was removed from the mold and cut with a razor blade, such that the distance from the sample holes to the phantom’s edges was 1.0 cm. To image samples, sample solutions were pipetted into FEP tubing (0.08″ diameter, cut to 10-cm long), which was inserted into the phantom and sealed by folding over the ends and securing with additional tubing (0.12″ diameter, cut to 5-mm long). Images were acquired using the Step and Shoot mode with 120 angles and 10 pulses per angle. Data were analyzed using OsiriX Lite software (Version 8.0). Thick slab processing was used to visualize accumulated signal over 5 mm. ROIs of equal area were drawn around areas containing signal, and mean PA signal in each ROI was recorded.

### PA imaging in vivo

Prior to imaging, hair was removed from the lower body half by shaving and treating with a topical hair removal cream. Mice were positioned on their sides such that the area to be imaged was directly above the light source. Images were acquired using the Step and Shoot mode with 120 angles and 30 pulses per angle. Data were analyzed using OsiriX software. Thick slab processing was used to visualize accumulated signal over 12 mm. ROIs of equal area were drawn to include all visible signal in the image, and mean PA signal in each ROI was recorded. Change in PA signal was calculated by subtracting signal from images taken prior to injection from those at given time points.

### Statistical analyses

Statistical analyses were performed in GraphPad Prism version 6.0c. Sample sizes in all experiments were sufficiently powered to detect at least a *p* value < 0.05, which was considered to be significant. All data were analyzed using Student’s *t* tests. Data are expressed as mean ± SD. Group variances were similar in all cases.

### Synthetic methods

Experimental procedures for the synthesis of HyP-1 and red-HyP-1 can be found in the Supplementary Methods. For NMR spectra of synthetic intermediates, see Supplementary Figs. 15–17. For NMR spectra of HyP-1 and red-HyP-1, see Supplementary Figs. 18–21.

### Data availability

The authors declare that all relevant data supporting the findings of this study are available within the article and in the Supplementary Information document, or from the corresponding author on request.

## Electronic supplementary material

Supplementary Information

Description of Additional Supplementary Files

Supplementary Movie 1
