# Supplementary material for: A bioreducible N-oxide-based probe for photoacoustic imaging of hypoxia
Source: Nat Commun. 2017 Nov 27;8:1794. doi: 10.1038/s41467-017-01951-0 (PMC5702603; doi:10.1038/s41467-017-01951-0)
Supplement: Supplementary file 1 — Supplementary Information [file 41467_2017_1951_MOESM1_ESM.pdf]

Supplementary Methods

Chemical and instruments

4-Aminoacetophenone was purchased from AK Scientific. Ethyl iodide, DIPEA, K_2CO_3, KOH, Na_2SO_4 and NH_4OAc were purchased from Oakwood Chemicals. Benzaldehyde, $BF_3{\cdot}OEt_2$, BIPY, DPI, DMSO, *m*-CPBA, nitroreductase and rat liver microsomes were purchased from Sigma Aldrich. Nitromethane was purchased from Alfa Aesar. Anaero-Indicators, AnaeroPack™ anaerobic gas generators, acetonitrile, *n*-BuOH, CH_2Cl_2, EtOAc and Hexanes were purchased from Fisher Scientific. $CDCl_3$ was purchased from Cambridge Isotopes Laboratory. Agarose LE was purchased from Gold Biotechnology. NADPH was purchased from EMD Millipore. All chemicals were used as purchased without further purification. Fluorinated ethylene propylene (FEP) tubing (wall thickness 0.01”, inner diameters 0.08” and 0.12”) were purchased from McMaster-Carr. Thin-layer chromatography (TLC) was performed using glass-backed TLC plates coated with silica gel containing an UV254 fluorescent indicator (Macherey-Nagel). Flash chromatography was performed with 230-400 mesh silica gel P60 (SiliCycle Inc). UV-visible spectra were recorded on a Cary 60 spectrometer. Fluorescence spectra were recorded on a QuantaMaster-400 scanning spectrofluorometer using micro fluorescence quartz cuvettes (Science Outlet). NMR spectra were taken using Bruker or Varian 500 MHz spectrometers at 25 °C. High-resolution mass spectra were acquired using Waters Q-Tof Ultima ESI and Waters Synapt G2-Si ESI/LC-MS spectrometers. Cellular imaging was performed using an EVOS FL cell imaging system. *In vivo* Fluorescence imaging was performed using an IVIS Spectrum CT live-animal imaging system (Perkin-Elmer, USA). PA imaging was performed using an Endra Nexus 128 photoacoustic tomography system (Ann Arbor, MI, USA).

Data Analysis

PA images were analysed using OsiriX software (version 8.0). Reported values correspond to mean PA signals in regions of interest (ROIs) of equal area. IVIS Spectrum images were analysed using Living Image software (version 4.1). Reported values correspond to total radiant efficiency in ROIs of equal area. Statistical calculations (Student’s T-test) were performed using GraphPad Prism (version 6.0c).

Buffer preparation

0.1 M potassium phosphate buffer (pH 7.4) was prepared by combining stock solutions of KH_2PO_4 (1 M, 0.990 mL) and K_2HPO_4 (1 M, 4.010 mL) and diluting to a final volume of 50 mL. 20 mM Britton-Robinson (BRB) buffers (pH 4.0 – 12.0, 50% v/v EtOH) were prepared as described[1]. Fine adjustments to pH were made via addition of 0.1 M HCl or 0.1 M NaOH. pH values were determined using a Mettler-Toledo SevenCompact pH meter calibrated using pH 4.0, 7.0 and 10.0 standard buffers at 25 °C.

***In vivo* injection formulations**

For all *in vivo* experiments, HyP-1 was formulated in sterile saline containing 10% DMSO. Sterile saline was prepared by dissolving NaCl (90 mg) in Milli-Q H_2O (10 mL) and filtering the resulting solution through a Millex-GS 0.22 μM sterile filter.

Synthetic Methods

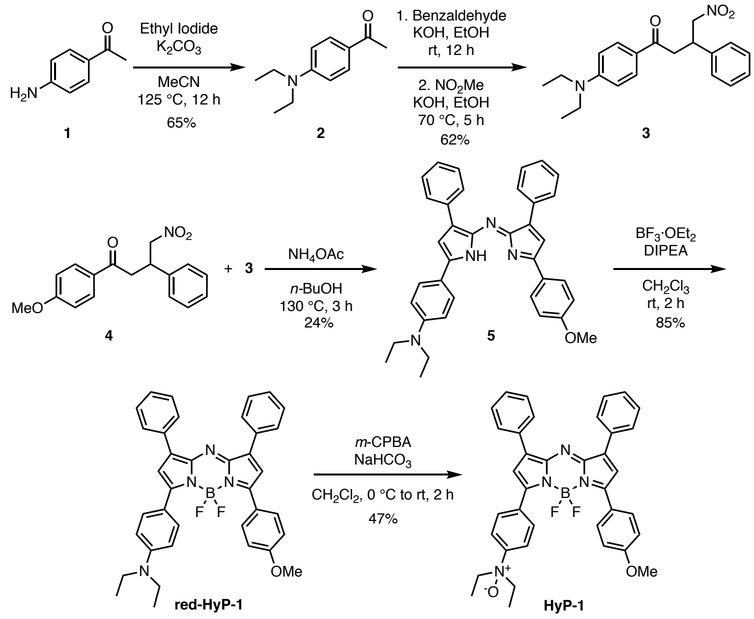

1-(4-(Diethylamino)phenyl)ethan-1-one **(2)**. To a solution of 4-aminoacetophenone **(1)** (1.35 g, 10.0 mmol) in MeCN (20 mL) was added ethyl iodide (3.20 mL, 4 equiv.) and K_2CO_3 (2.76 g, 2 equiv.). The mixture was heated to 125 °C in a sealed pressure flask overnight, cooled to room temperature and filtered. The filtrate was concentrated and purified by column chromatography (20% EtOAc/Hexanes) to afford the desired compound (1.25 g, 65%) as an off-white solid. ^1H NMR (500 MHz, $CDCl_3$) δ 7.85 (d, J = 8.6 Hz, 2H), 6.62 (d, J = 8.8 Hz, 2H), 3.42 (q, J = 7.1 Hz, 4H), 2.49 (s, 3H), 1.20 (t, J = 6.9 Hz, 6H). ^{13}C NMR (125 MHz, $CDCl_3$) δ 196.30, 151.36, 131.07, 124.92, 110.33, 44.76, 26.13, 12.75.

1-(4-(Diethylamino)phenyl)-4-nitro-3-phenylbutan-1-one **(3)**. To a solution of **2** (9.0 g, 47.2 mmol) in EtOH (236 mL) was added benzaldehyde (4.8 mL, 1 equiv.) and aqueous KOH (10 M, 14.2 mL, 3 equiv.). The reaction was stirred overnight at room temperature. Nitromethane (28.8 mL, 10 equiv.) was added and the reaction was stirred at reflux for 5 hours. The mixture was cooled to room temperature, concentrated, re-dissolved in EtOAc and washed with brine. The organic layer was dried over Na_2SO_4, concentrated and purified by column chromatography (20% EtOAc/Hexanes) to provide the desired compound (9.96 g, 62%) as an off-white solid. ^1H NMR (500 MHz, $CDCl_3$) δ 7.85 (d, J = 9.1 Hz, 2H), 7.36 – 7.24 (m, 5H), 6.63 (d, J = 9.2 Hz, 2H), 4.87 (dd, J = 12.6, 5.9 Hz, 1H), 4.68 (dd, J = 12.6, 8.9 Hz, 1H), 4.24 (tt, J = 8.6, 5.9 Hz, 1H), 3.42 (q, J = 7.1 Hz, 4H), 3.37 (dd, J = 17.0, 6.0 Hz, 1H), 3.29 (dd, J = 17.1, 8.2 Hz, 1H), 1.21 (t, J = 7.1 Hz,

6H). ^{13}C NMR (125 MHz, $CDCl_3$) δ 194.57, 151.71, 140.09, 130.90, 129.19, 127.87, 127.78, 123.86, 110.48, 80.03, 44.82, 40.96, 40.00, 12.76.

(Z)-N,N-diethyl-4-(5-((5-(4-methoxyphenyl)-3-phenyl-2H-pyrrol-2-ylidene)amino)-4-phenyl-1H-pyrrol-2-yl)aniline **(5)**. To a suspension of **4**[2] (1.05 g, 3.08 mmol) in *n*-BuOH (60 mL) was added **3** (1.85 g, 2 equiv.). The mixture was heated at 70 °C until the solids were completely dissolved. NH_4OAc (3.57 g, 15 equiv.) was added, and the mixture was refluxed for 3 hours. The resulting dark green solution was cooled to room temperature, concentrated, dissolved in CH_2Cl_2 and washed with brine. The organic layer was separated, and the aqueous layer was back extracted with CH_2Cl_2 (×3). The combined organic layers were dried with Na_2SO_4 and concentrated. The mixture was purified by column chromatography (20-40% CH_2Cl_2/Hexanes) to afford the desired product (410 mg, 24%) as a blue-green solid. ^1H NMR (500 MHz, $CDCl_3$) δ 8.13 – 8.06 (m, 4H), 7.95 (d, J = 9.0 Hz, 2H), 7.78 (d, J = 8.8 Hz, 2H), 7.46 – 7.38 (m, 4H), 7.38 – 7.33 (m, 1H), 7.33 – 7.27 (m, 2H), 7.05 (d, J = 8.8 Hz, 2H), 6.97 (s, 1H), 6.79 (d, J = 9.0 Hz, 2H), 3.91 (s, 3H), 3.49 (q, J = 7.1 Hz, 4H), 1.27 (t, J = 7.0 Hz, 6H). ^{13}C NMR (125 MHz, $CDCl_3$) δ 163.38, 160.31, 150.19, 145.97, 144.34, 143.64, 141.90, 136.53, 134.89, 134.06, 129.83, 129.44, 128.98, 128.38, 128.37, 128.18, 127.26, 127.13, 125.16, 119.70, 118.83, 114.85, 111.81, 109.91, 55.71, 44.88, 12.98. HR-MS calcd for $C_{37}H_{35}N_4O^+$ $[M+H]^+$ 551.2811, found 551.2800.

red-HyP-1. To a solution of **5** (53.0 mg, 0.096 mmol) in anhydrous CH_2Cl_2 (2.0 mL) was added DIPEA (0.168 mL, 10 equiv.) followed by $BF_3{\cdot}OEt_2$ (0.177 mL, 15 equiv.). The reaction was stirred at room temperature for 2 hours, then quenched with the addition of saturated $NaHCO_3$. The organic layer was separated, and the aqueous layer was back extracted with CH_2Cl_2 (×3). The combined organic layers were dried over Na_2SO_4 and concentrated. The crude solid was purified by column chromatography (40-50% CH_2Cl_2/Hexanes) to afford the desired product (48.9 mg, 85%) as a dark pink solid. ^1H NMR (500 MHz, $CDCl_3$) δ 8.21 (d, *J* = 9.2 Hz, 2H), 8.13 – 8.02 (m, 6H), 7.52 – 7.41 (m, 5H), 7.40 – 7.33 (m, 1H), 7.27 (s, 1H), 7.02 (d, *J* = 8.9 Hz, 2H), 6.92 (s, 1H), 6.75 (d, *J* = 9.3 Hz, 2H), 3.89 (s, 3H), 3.47 (q, *J* = 7.1 Hz, 4H), 1.25 (t, *J* = 7.2 Hz, 6H). ^{13}C NMR (125 MHz, $CDCl_3$) δ 160.94, 160.17, 152.69, 150.93, 147.18, 143.69, 143.56, 138.80, 133.77, 133.39, 132.61, 131.19, 129.55, 129.43, 129.17, 128.70, 128.64, 128.37, 125.75,

120.16, 117.32, 116.77, 114.18, 111.84, 55.58, 45.00, 12.99. HR-MS calcd for $C_{37}H_{34}BF_2N_4O^+$ $[M+H]^+$ 599.2794, found 599.2795.

HyP-1. A solution of **red-HyP-1** (71.3 mg, 0.119 mmol) in CH_2Cl_2 (2.4 mL) was cooled to 0 °C in an ice bath. $NaHCO_3$ (30 mg, 1.1 equiv.) and *m*-CPBA (77% w/w, 80 mg, 1.1 equiv.) were added, and the mixture was warmed to room temperature and stirred for 2 hours. The mixture was then poured into a solution of saturated $NaHCO_3$ and extracted with EtOAc (×3). The organic layers were combined, and the solvent was removed to yield a dark green film. The crude product was purified by column chromatography to afford the desired product (15 mg, 47%) as a dark green solid. ^{1}H NMR (500 MHz, $CDCl_3$) δ 8.08 (dd, *J* = 9.0, 2.7 Hz, 4H), 7.99 (td, *J* = 8.4, 1.6 Hz, 4H), 7.83 (d, *J* = 8.7 Hz, 2H), 7.43 – 7.31 (m, 6H), 7.06 (s, 1H), 6.96 (d, *J* = 9.0 Hz, 2H), 6.94 (s, 1H), 3.82 (s, 3H), 3.81 – 3.68 (m, 4H), 1.13 (t, *J* = 7.0 Hz, 6H). ^{13}C NMR (125 MHz, $CDCl_3$) δ 170.05, 162.85, 161.42, 154.13, 146.74, 145.35, 144.45, 142.15, 132.58, 132.23, 131.87, 130.07, 129.87, 129.46, 129.22, 129.19, 128.67, 128.62, 123.33, 122.03, 119.96, 118.01, 117.98, 114.52, 66.84, 55.55, 29.71. HR-MS calcd for $C_{37}H_{34}BF_2N_4O_2^+$ $[M+H^+]$ 615.2737, found 615.2742.

Compound	ε ($M^{-1}\cdot cm^{1}$)	λ_{abs} (nm)	λ_{em} (nm)	Φ_F
HyP-1	1.4×10^4	670	697	0.33
Red-HyP-1	5.4×10^4	760	798	0.15

Supplementary Table 1. Photophysical properties of HyP-1 and Red-HyP-1 measured in $CHCl_3$.

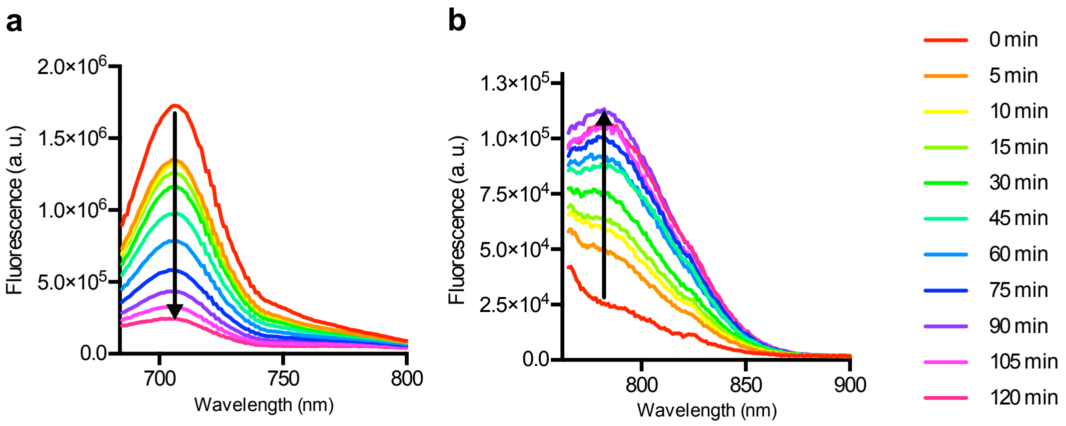

Supplementary Figure 1. (a) Time-dependent fluorescence decrease at 710 nm and (b) time-dependent fluorescence increase at 785 nm corresponding to conversion of HyP-1 to red-HyP-1 by rat liver microsomes under hypoxic conditions. HyP-1 (2 μM), rat liver microsomes (10 μL) and NADPH (50 μM) were incubated at 37 °C in degassed 0.1 M potassium phosphate buffer (pH 7.4) under hypoxic conditions, and fluorescence spectra were recorded at the indicated time points. HyP-1 was excited at 672 nm and emission was collected from 685 to 800 nm. Red-HyP-1 was excited at 750 nm and emission was collected from 760 to 900 nm.

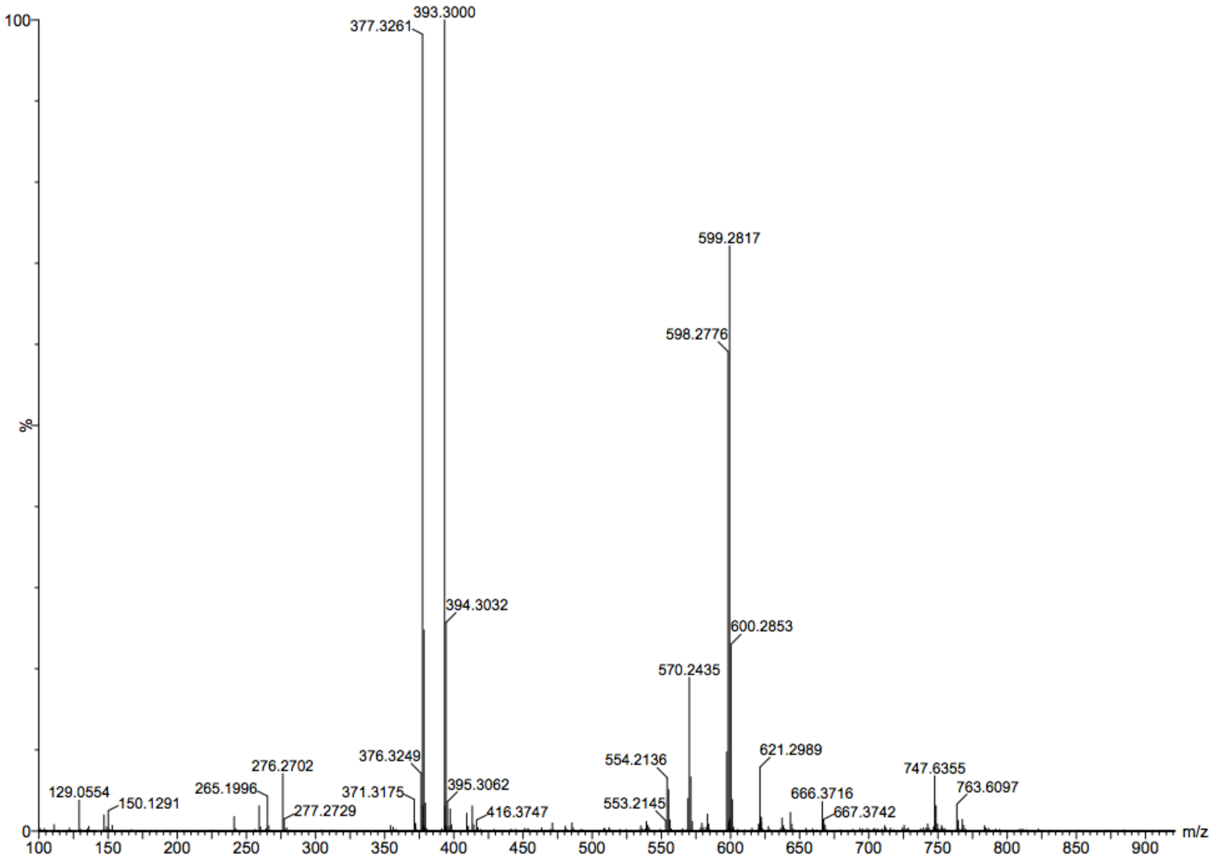

Supplementary Figure 2. Mass spectrum obtained from LC/MS analysis of reaction mixture containing HyP-1 (2 μM), rat liver microsomes (10 μL) and NADPH (50 μM) in 0.1 M potassium phosphate buffer (pH 7.4) after incubation under hypoxic conditions for 2 h. The reaction mixture was quenched upon addition of 1 mL MeCN, vortexed, then centrifuged at 3000 rpm for 5 minutes. The supernatant was removed and analyzed via LC/MS. M/Z of 599.2817 corresponds to red-HyP-1 $[M+H]^+$.

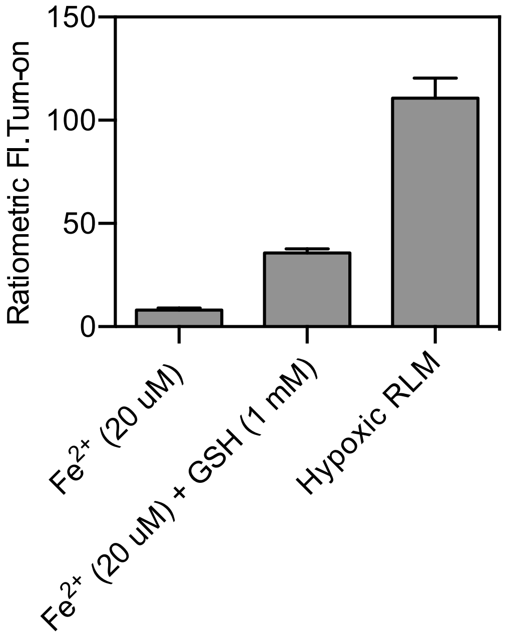

Supplementary Figure 3. Ratiometric fluorescence turn-on response of HyP-1 (2 μM) in the presence of $FeSO_4$ (20 μM) and $FeSO_4$ (20 μM) + GSH (1 mM) in 50 mM HEPES buffer (pH 7.4, 0.1% v/v CrEL) after 1 h incubation at 37 °C. Turn-on response in the presence of rat liver microsomes under hypoxic conditions shown for comparison.

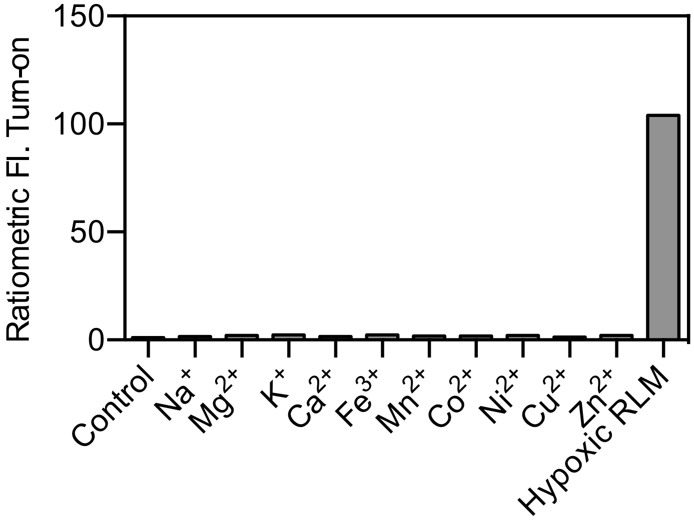

Supplementary Figure 4. Ratiometric fluorescent turn-on response of HyP-1 (2 μM) in the presence of various metal ions in 0.1 M potassium phosphate buffer (pH 7.4, 0.1% v/v CrEL) after 2 h incubation at 37 °C. Concentrations used were 2 mM for Na^{+}, Mg^{2+}, K^{+} and Ca^{2+}, and 50 μM for all other metal ions.

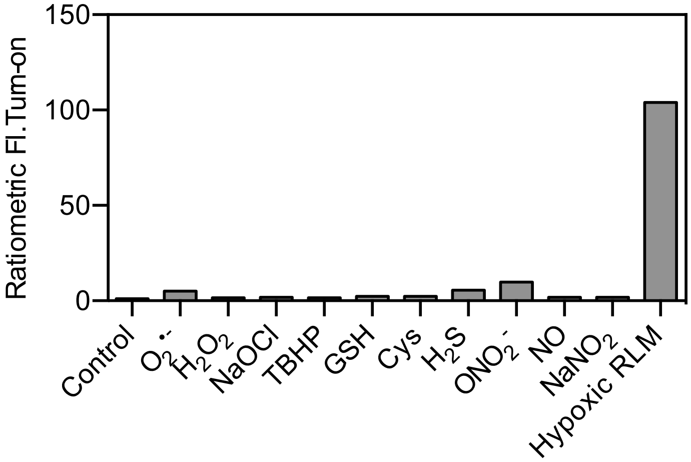

Supplementary Figure 5. Ratiometric fluorescence turn-on response of HyP-1 (2 μM) in the presence of various reactive oxygen, nitrogen and sulfur species (100 μM) in 0.1 M potassium phosphate buffer (pH 7.4, 0.1% v/v CrEL) after 2 h incubation at 37 °C.

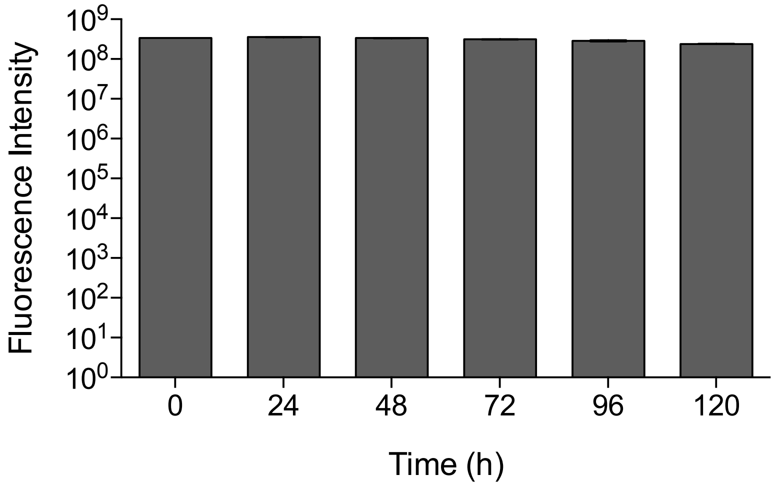

Supplementary Figure 6. Fluorescence intensity (Ex. = 670 nm) of HyP-1 (2 μM) at the indicated time points during extended incubation in 0.1 M potassium phosphate buffer (pH 7.4, 0.1% v/v CrEL) under ambient light and temperature. Represented as mean ± SD (n = 3).

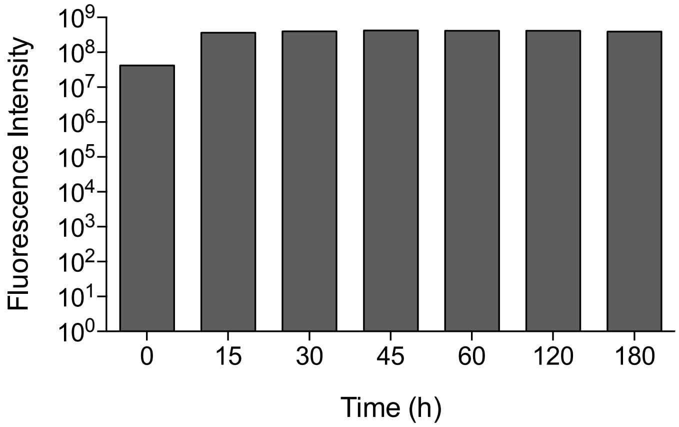

Supplementary Figure 7. Fluorescence intensity (Ex. = 670 nm) of HyP-1 (2 μM) at the indicated time points during incubation in 0.1 M potassium phosphate buffer (pH 7.4) supplemented with human plasma (20% v/v) at 37 °C for 3 h.

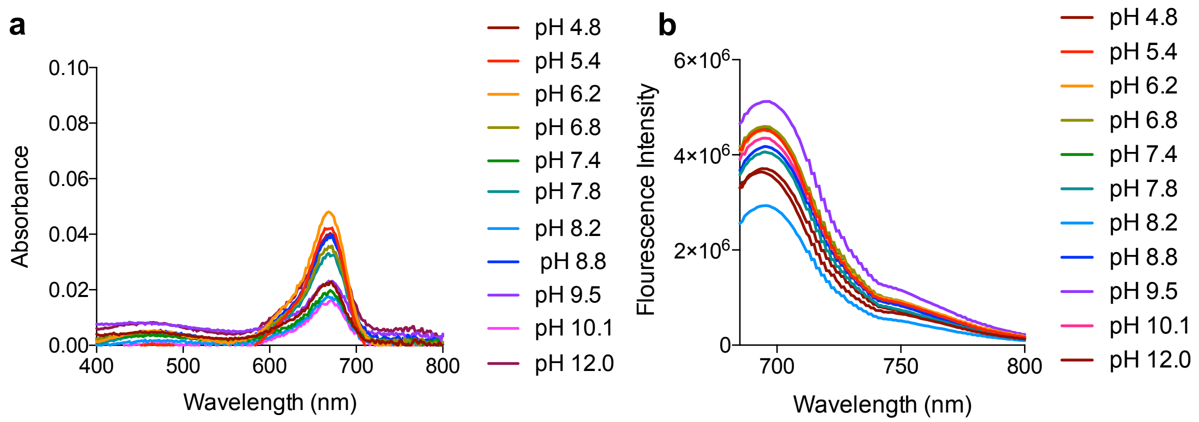

Supplementary Figure 8. (a) Absorbance and (b) fluorescence spectra of HyP-1 (2 μM) in 20 mM BRB buffers containing 50% v/v EtOH (pH range 4.8-12.0).

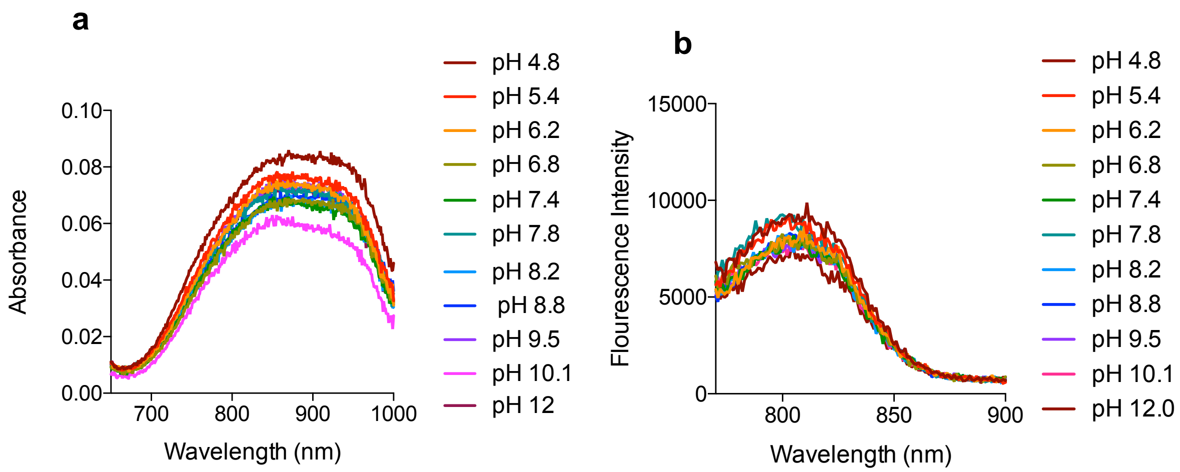

Supplementary Figure 9. (a) Absorbance and (b) fluorescence spectra of red-HyP-1 (2 μM) in 20 mM BRB buffers containing 50% v/v EtOH (pH range 4.8-12.0).

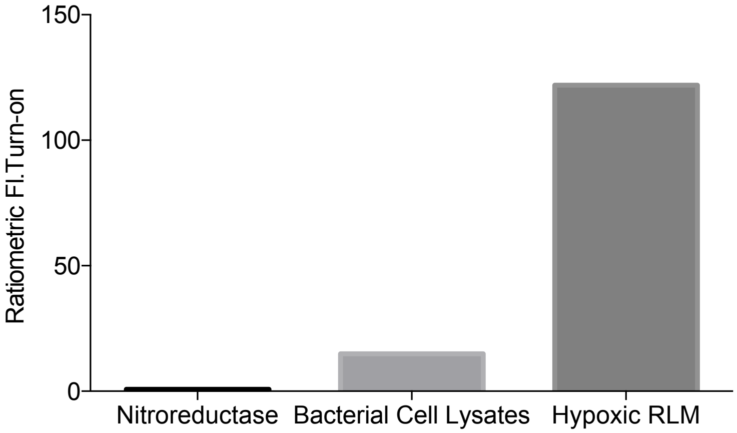

Supplementary Figure 10. Ratiometric fluorescence turn-on of HyP-1 in the presence of purified nitroreductase enzyme and bacterial cell lysates in comparison to turn-on observed in the presence of rat liver microsomes under hypoxic conditions. For the nitroreductase experiment, HyP-1 (2 μM) was incubated in the presence of nitroreductase (0.5 μg/mL) and NADH (100 μM) in 0.1 M potassium phosphate buffer (pH 7.4) at 37 °C for 2 h. For the bacterial experiment, bacteria were grown overnight and cells were lysed via sonication. The suspension was centrifuged at 4000 rpm for 10 min, and the supernatant was removed. HyP-1 (2 μM) was incubated with cell lysate solution (1 mL) at 37 °C for 2 h.

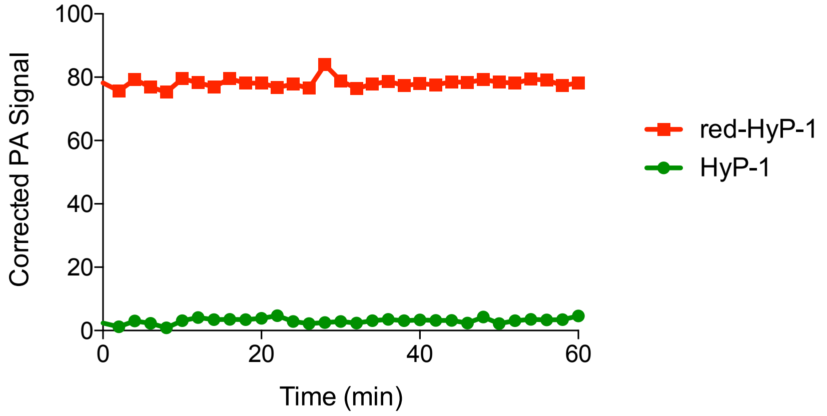

Supplementary Figure 11. PA signal (770 nm) at the indicated time points of HyP-1 (green) and red-HyP-1 (red) solutions (10 μM in 0.1 M potassium phosphate buffer with 50% v/v EtOH) produced upon continuous image acquisition over the course of 1 h. Reported values represent mean PA signal observed after subtracting background signal produced from tissue-mimicking phantom.

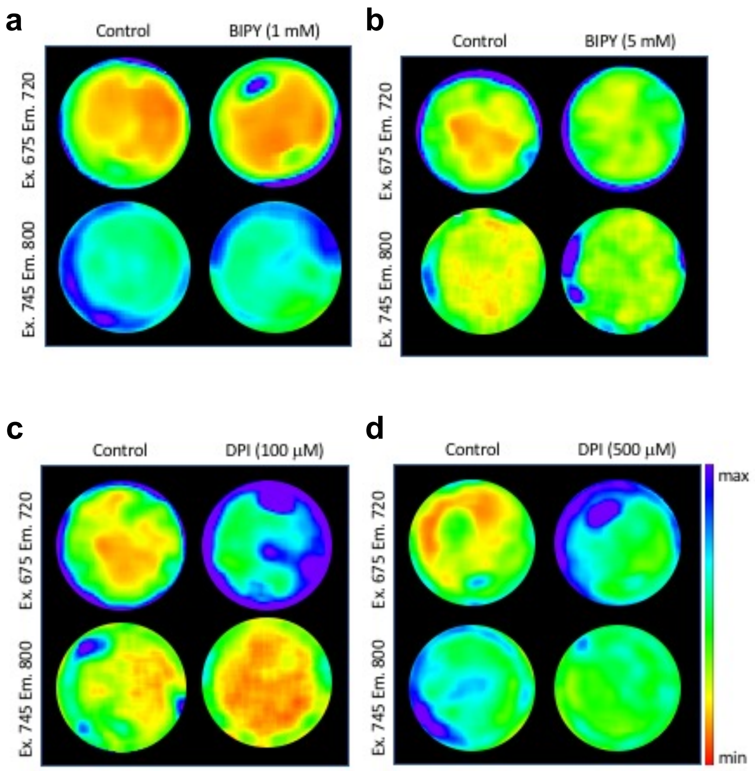

Supplementary Figure 12. Representative IVIS spectrum images of 4T1 cells plated on 6-well plates and treated with serum-free medium containing HyP-1 (5 μM) and (a) BIPY (1 mM) (b) BIPY (5 mM) (c) DPI (100 μM) and (d) DPI (500 μM). Cells were incubated under hypoxic conditions for 4 hours prior to imaging. Images were acquired using 675/720 and 745/800 excitation/emission filter sets to visualize HyP-1 and red-HyP-1, respectively. A vehicle control for each condition is shown for comparison.

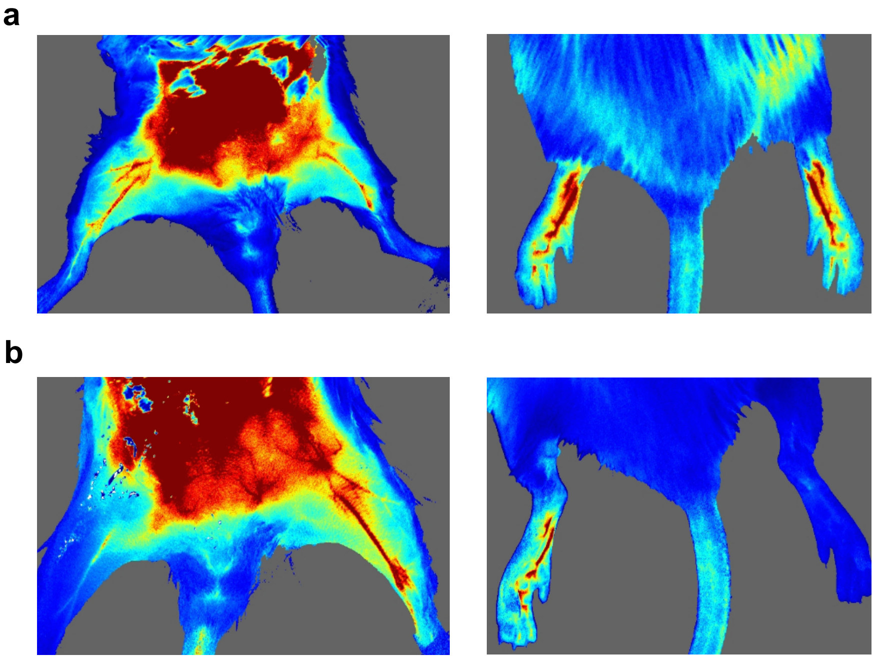

Supplementary Figure 13. Laser Doppler perfusion images acquired (a) before and (b) 15 minutes after surgical ligation of the femoral artery in the right hindlimb.

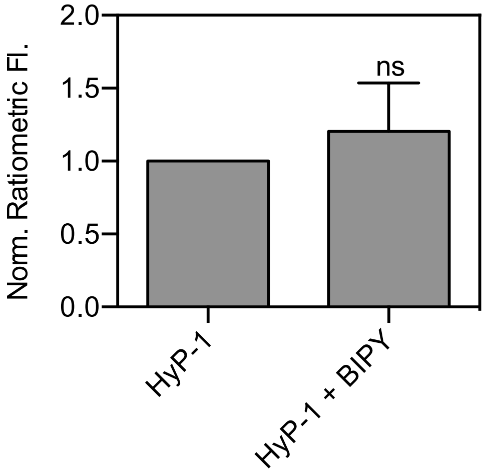

Supplementary Figure 14. Normalized ratiometric fluorescence of ischemic hindlimbs following intramuscular injection of HyP-1 (50 μL, 50 μM) or HyP-1 + BIPY (5 mM). Surgical ligation of the femoral artery was performed on both hindlimbs of female BALB/c mice (6 weeks old). HyP-1 was injected intramuscularly into one of the hindlimbs, while a HyP-1 solution containing BIPY was injected into the other hindlimb. Images were acquired using 675/720 nm and 745/800 nm filter sets. Ratiometric fluorescence of the BIPY-treated hindlimb was normalized with respect to the control. Results are presented as mean ± SD (n = 3).

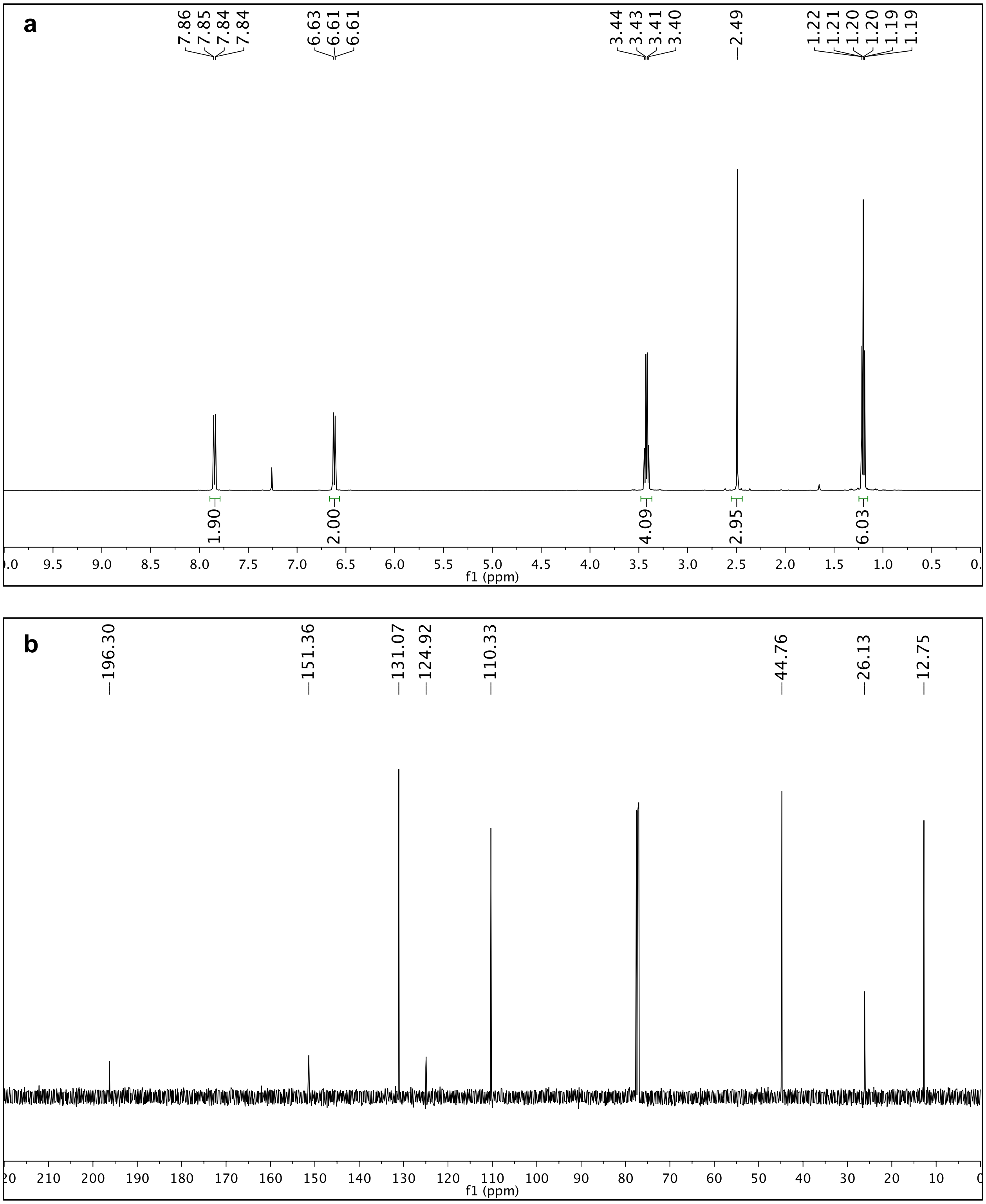

Supplementary Figure 15. (a) ^{1}H NMR (500 MHz, $CDCl_3$) and (b) ^{13}C NMR (125 MHz, $CDCl_3$) spectra of **2**.

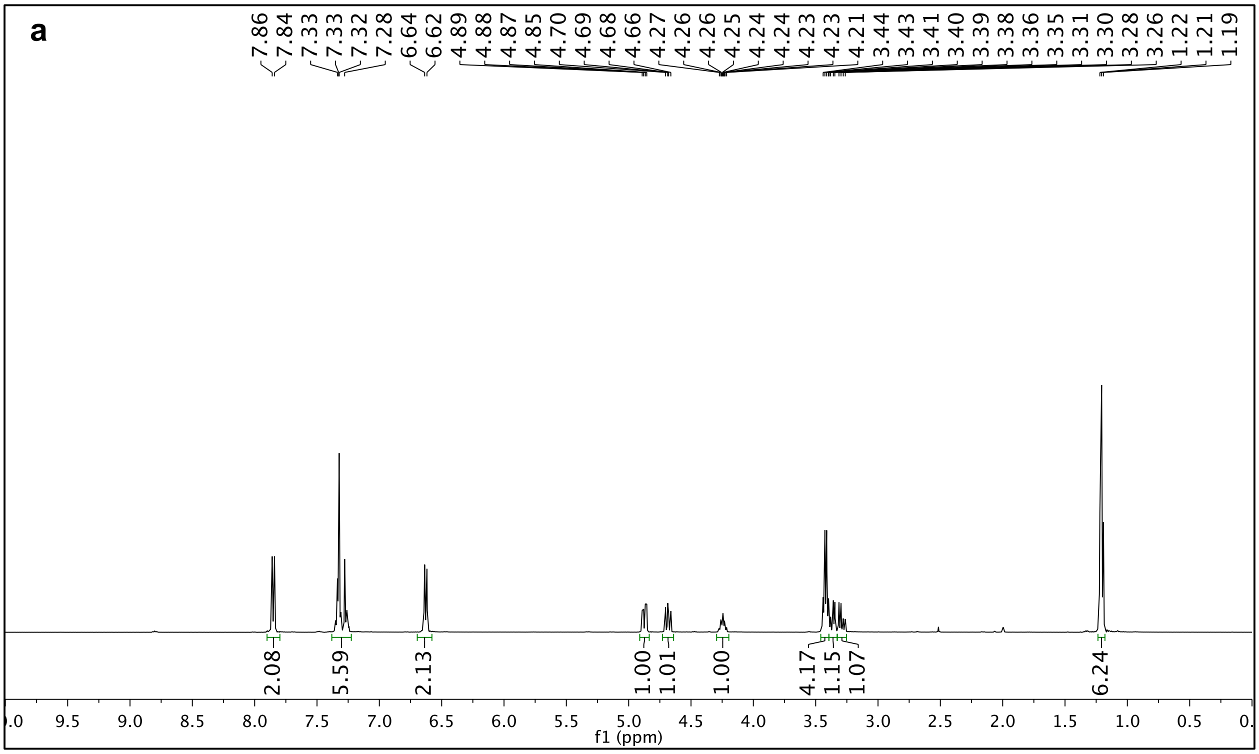

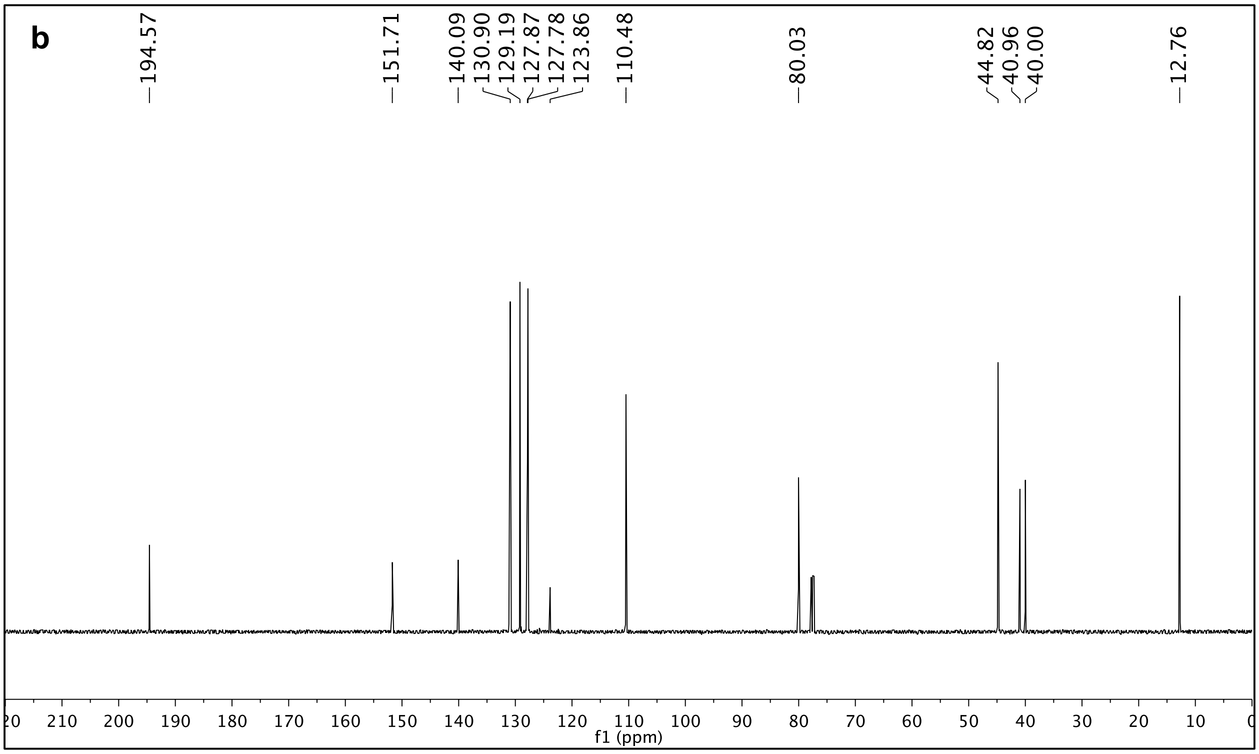

Supplementary Figure 16. (a) ^{1}H NMR (500 MHz, $CDCl_3$) and (b) ^{13}C NMR (125 MHz, $CDCl_3$) spectra of **3**.

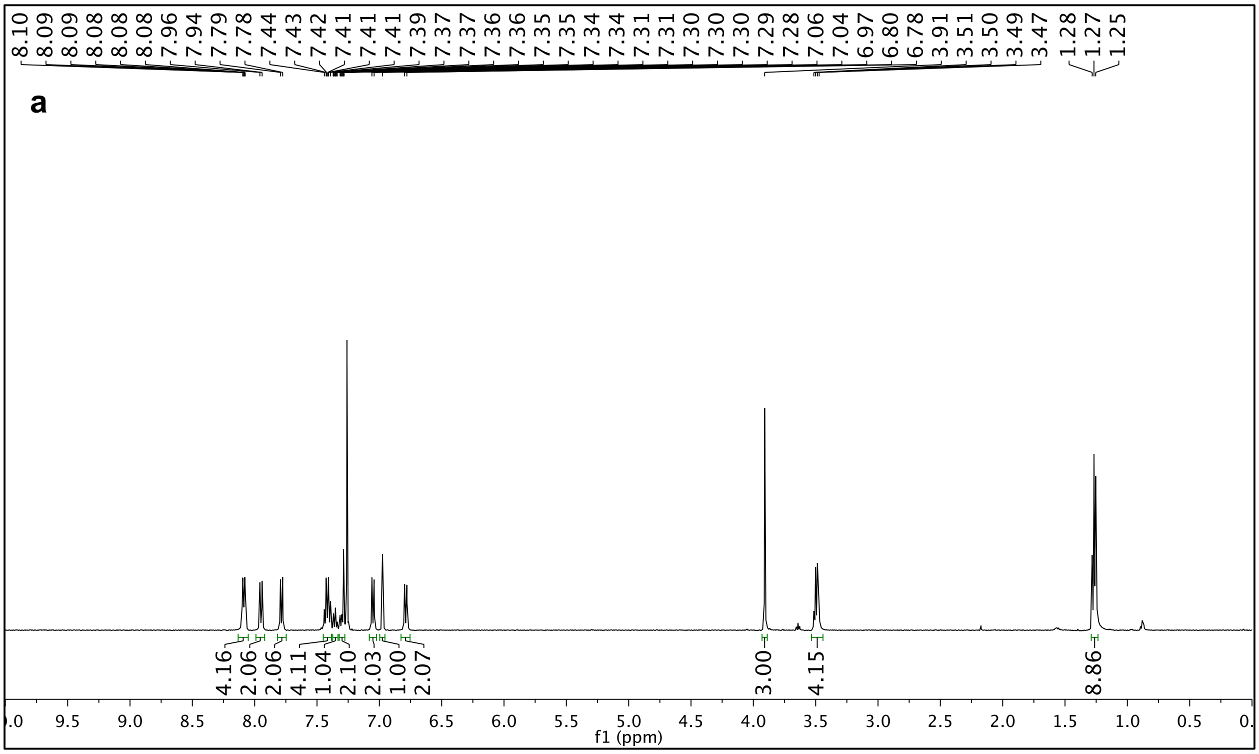

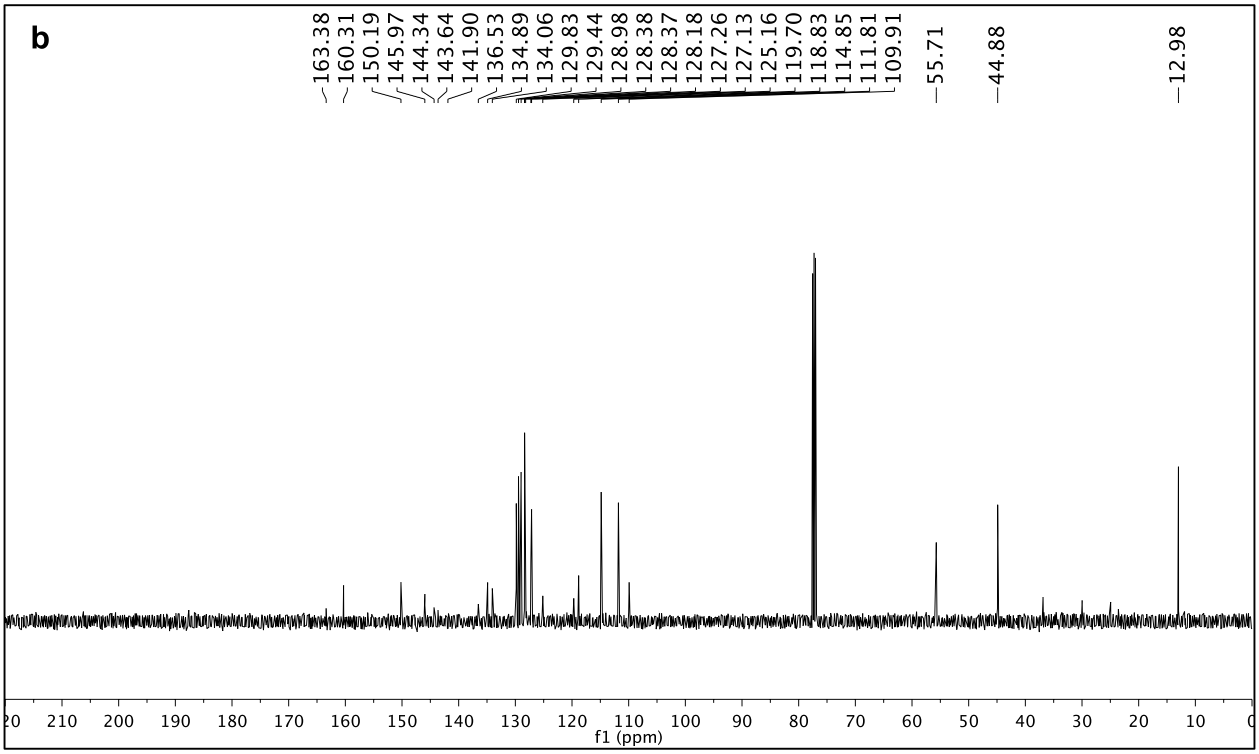

Supplementary Figure 17. (a) ^{1}H NMR (500 MHz, $CDCl_3$) and (b) ^{13}C NMR (125 MHz, $CDCl_3$) spectra of **5**.

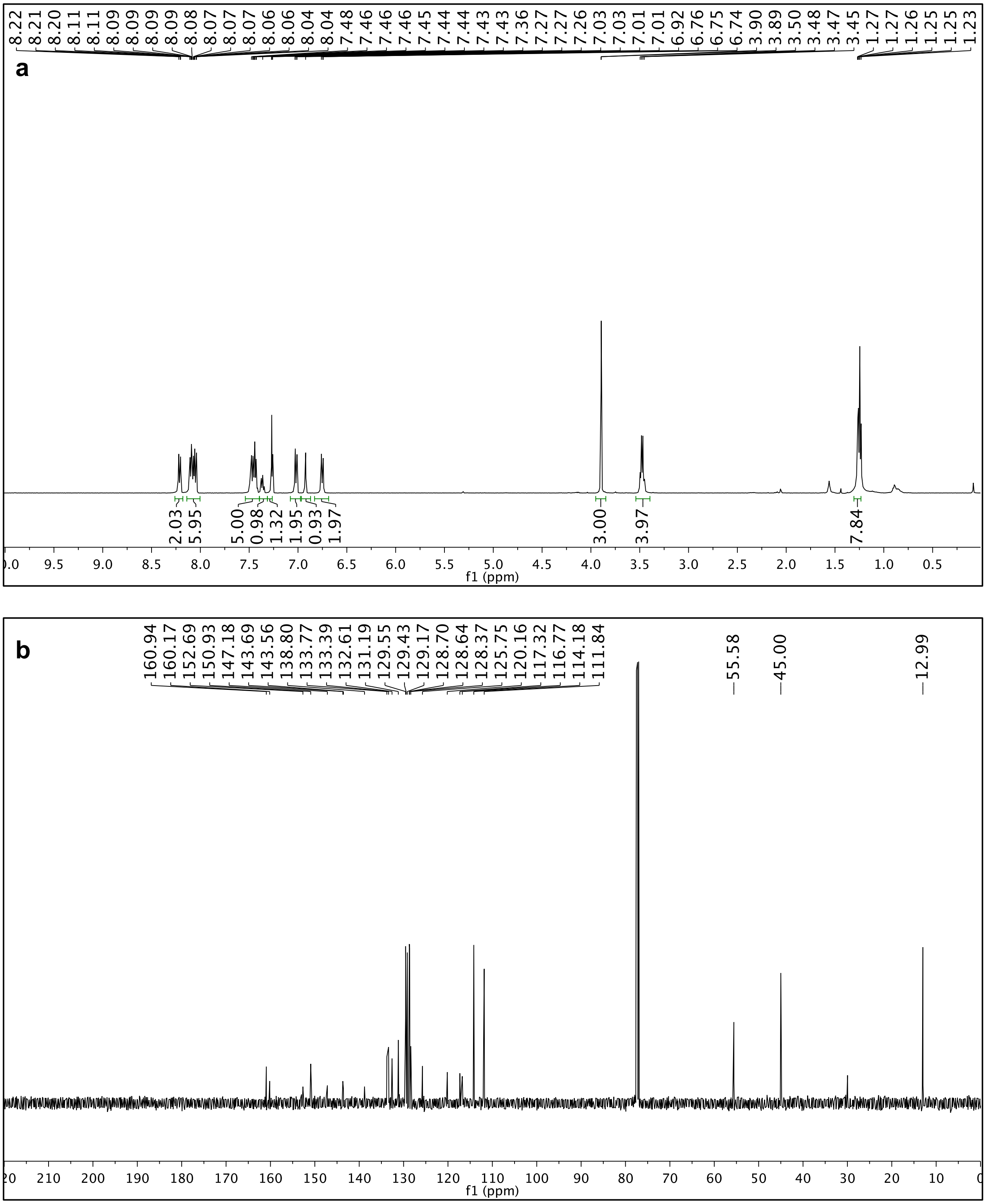

Supplementary Figure 18. (a) ^{1}H NMR (500 MHz, $CDCl_3$) and (b) ^{13}C NMR (125 MHz, $CDCl_3$) spectra of **red-HyP-1**.

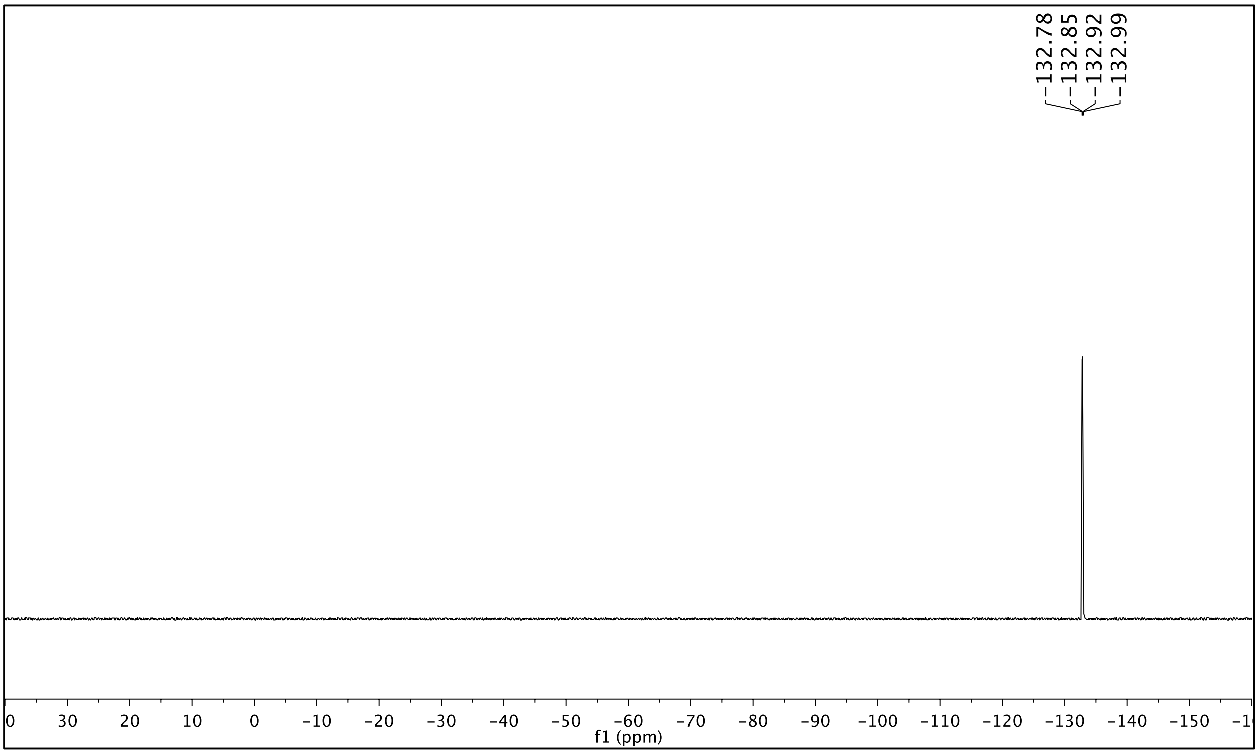

Supplementary Figure 19. ^{19}F NMR (470 MHz, $CDCl_3$) spectrum of **red-HyP-1.**

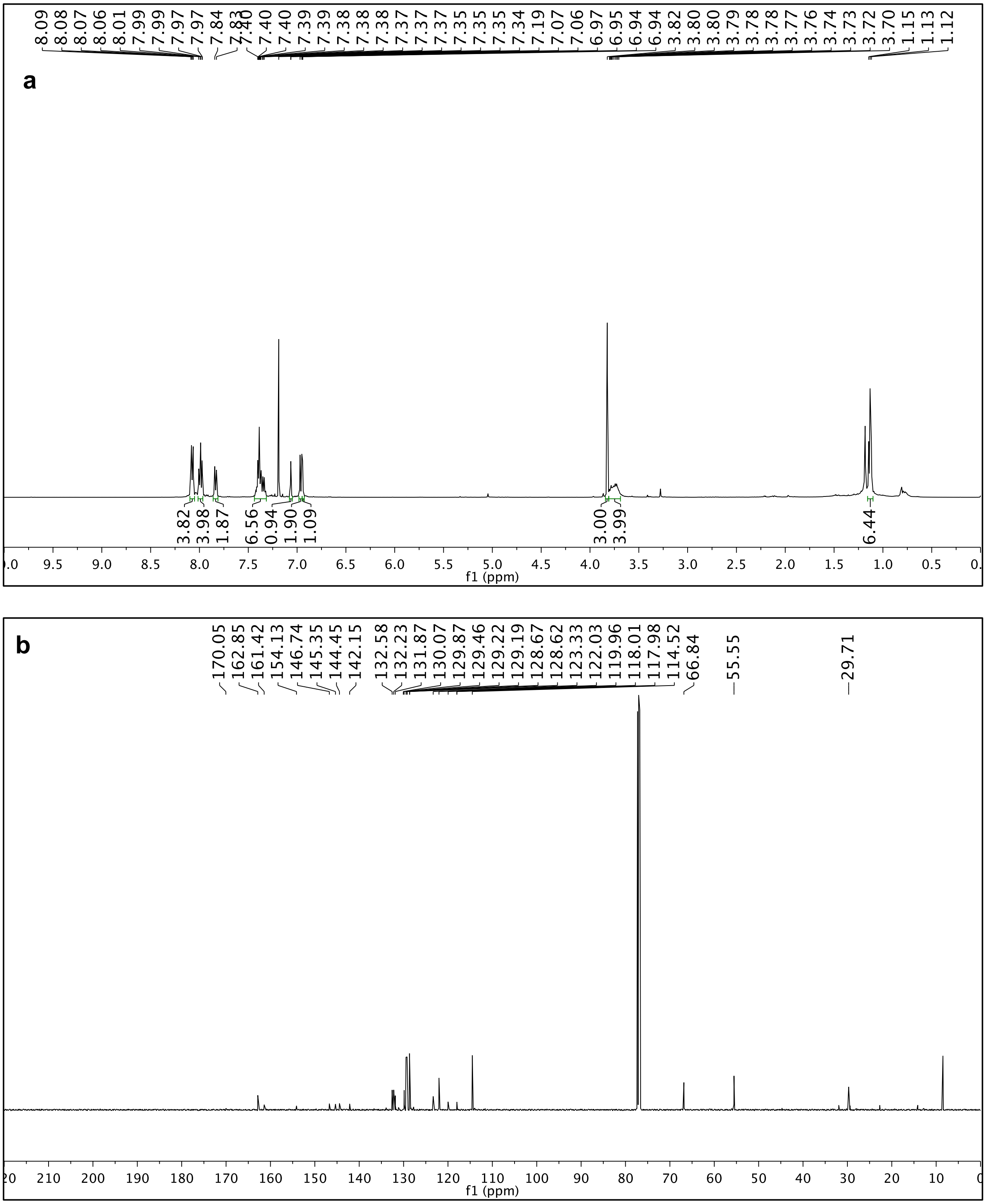

Supplementary Figure 20. (a) ^{1}H NMR (500 MHz, $CDCl_3$) and (b) ^{13}C NMR (125 MHz, $CDCl_3$) spectra of **HyP-1**.

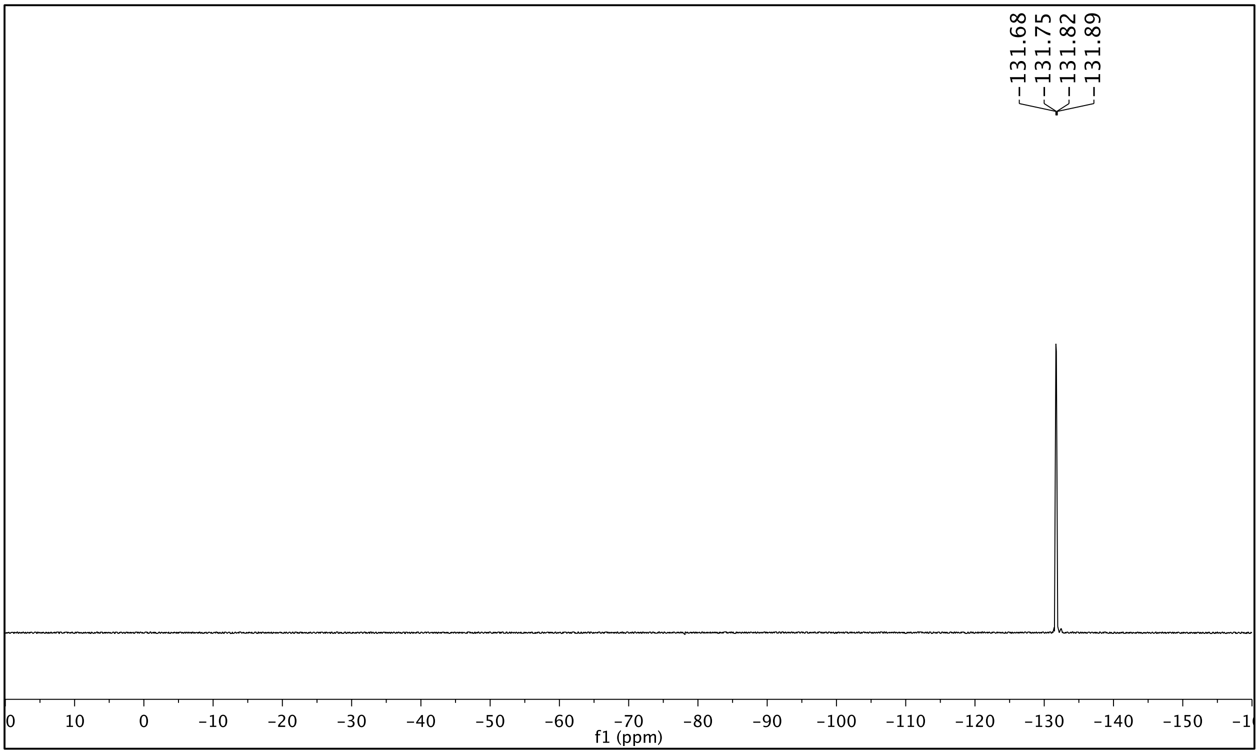

Supplementary Figure 21. ^{19}F NMR (470 MHz, $CDCl_3$) spectrum of **HyP-1.**

Supplementary References

1. Reynolds III, J. E. *et al.* Spectral and redox properties of the GFP synthetic chromophores as a function of pH in buffered media. *Chem. Commun.* **49,** 7788 (2013).
2. Gorman, A. *et al.* In vitro demonstration of the heavy-atom effect for photodynamic therapy. *J. Am. Chem. Soc.* **126,** 10619–10631 (2004).
