# Supplementary material for: A bioreducible N-oxide-based probe for photoacoustic imaging of hypoxia
Source: Nat Commun. 2017 Nov 27;8:1794. doi: 10.1038/s41467-017-01951-0 (PMC5702603; doi:10.1038/s41467-017-01951-0)
Supplement: Supplementary file 2 — Description of Additional Supplementary Files [file 41467_2017_1951_MOESM2_ESM.pdf]

Description of Additional Supplementary Files

File Name: Supplementary Movie 1
Description: 3D PA Reconstruction of in vivo imaging of a hypoxic tumor with HyP-1
